# A Randomized Controlled Trial of Cognitive Training Using a Visual Speed of Processing Intervention in Middle Aged and Older Adults

**DOI:** 10.1371/journal.pone.0061624

**Published:** 2013-05-01

**Authors:** Fredric D. Wolinsky, Mark W. Vander Weg, M. Bryant Howren, Michael P. Jones, Megan M. Dotson

**Affiliations:** 1 Department of Health Management and Policy, University of Iowa, Iowa City, Iowa, United States of America; 2 Center for Comprehensive Access and Delivery Research and Evaluation, Iowa City VA Health Care System, Iowa City, Iowa, United States of America; 3 Department of Biostatistics, University of Iowa, Iowa City, Iowa, United States of America; 4 College of Nursing, University of Iowa, Iowa City, Iowa, United States of America; Federal University of Rio de Janeiro, Brazil

## Abstract

**Background:**

Age-related cognitive decline is common and may lead to substantial difficulties and disabilities in everyday life. We hypothesized that 10 hours of visual speed of processing training would prevent age-related declines and potentially improve cognitive processing speed.

**Methods:**

Within two age bands (50–64 and≥65) 681 patients were randomized to (a) three computerized visual speed of processing training arms (10 hours on-site, 14 hours on-site, or 10 hours at-home) or (b) an on-site attention control group using computerized crossword puzzles for 10 hours. The primary outcome was the Useful Field of View (UFOV) test, and the secondary outcomes were the Trail Making (Trails) A and B Tests, Symbol Digit Modalities Test (SDMT), Stroop Color and Word Tests, Controlled Oral Word Association Test (COWAT), and the Digit Vigilance Test (DVT), which were assessed at baseline and at one year. 620 participants (91%) completed the study and were included in the analyses. Linear mixed models were used with Blom rank transformations within age bands.

**Results:**

All intervention groups had (*p*<0.05) small to medium standardized effect size improvements on UFOV (Cohen's *d* = −0.322 to −0.579, depending on intervention arm), Trails A (*d* = −0.204 to −0.265), Trails B (*d* = −0.225 to −0.320), SDMT (*d* = 0.263 to 0.351), and Stroop Word (*d* = 0.240 to 0.271). Converted to years of protection against age-related cognitive declines, these effects reflect 3.0 to 4.1 years on UFOV, 2.2 to 3.5 years on Trails A, 1.5 to 2.0 years on Trails B, 5.4 to 6.6 years on SDMT, and 2.3 to 2.7 years on Stroop Word.

**Conclusion:**

Visual speed of processing training delivered on-site or at-home to middle-aged or older adults using standard home computers resulted in stabilization or improvement in several cognitive function tests. Widespread implementation of this intervention is feasible.

**Trial Registration:**

ClinicalTrials.gov NCT-01165463

## Introduction

Age-related cognitive decline is common and affects memory, orientation, attention, abstract thinking, and perception [1]–[4]. These cognitive declines may lead to substantial difficulties and disabilities in everyday life [5]–[8]. Because life expectancy is at an all-time high and improving [9], identifying interventions that can be widely and efficiently implemented and that may prevent or even reverse cognitive decline are clinical and public health priorities [10]–[12]. This is especially important given evidence that cognitive declines are well-documented as early as age 30 in cross-sequential data [13]–[15] and as early as age 45 in longitudinal data [16].

Because part (but clearly not all) of these declines reflect negative brain plasticity, cognitive abilities may be strengthened somewhat by interventions that promote positive brain plasticity [7], [10], [17], [18]. Among the most promising such interventions are complex video games that train strategic control in structured situations [7]. Ball and Roenker [19]–[21] developed the precursor to such a video game intervention focusing on visual speed of processing. Their original training program was used as one of three interventions in the U.S. National Institutes of Health (NIH) funded, multi-site Advanced Cognitive Trial for Independent and Vital Elderly (ACTIVE), the largest cognitive training trial ever conducted [22]–[24]. Results from ACTIVE demonstrated that each of the three cognitive training interventions—memory, reasoning, and visual speed of processing—affected their targeted proximal and primary outcomes over both short- and long-term (1–5 year) follow-up periods [22]–[24] and reflected the equivalent of 6, 4, and 8 years, respectively, of cognitive decline restoration [25]. However, only the visual speed of processing intervention had significant and substantial effects on a variety of health outcomes including health-related quality of life, depressive symptoms and the onset of suspected clinical depression, self-rated health, and internal locus of control that lasted up to five years [26]–[32].

ACTIVE, however, had some limitations [33], [34]. First, because ACTIVE used a no contact rather than an attention control group, placebo effects could not be ruled out except by direct comparison of one training intervention to another. Second, because ACTIVE's booster training was compliance-conditioned, treatment effects could not be separated from adherence effects. Third, because ACTIVE relied on only one speed of processing outcome (the Useful Field of View test; UFOV [19]), which was thematically similar to the original visual speed of processing intervention, the possibility of “training to the test” existed. Fourth, because ACTIVE used Ball and Roenker's [19]–[21] original, MS-DOS based visual speed of processing intervention that required ongoing, supervised assistance and touch screen monitors, the potential for widespread implementation was limited. Finally, because ACTIVE only included participants≥65, it is not known whether the same training effects could be achieved at younger ages.

The objective of the Iowa Healthy and Active Minds Study (IHAMS) was to address these limitations. We designed IHAMS as a four-arm, parallel RCT that used the newest (MS-Windows based) version of the visual speed of processing training program that can be used on any personal computer (PC) without supervision. Participants were randomized separately within two age bands (50–64 and 65 years old or older) to either an attention control group using a computerized crossword puzzle game in a university-based laboratory, or to two active intervention groups receiving the visual speed of processing training in a university-based laboratory—one with and one without booster training, or to one active intervention group that was given the visual speed of processing software to take home and use on their own PCs. Primary and secondary neuropsychological outcomes were assessed at baseline and one year later.

## Methods

### Ethics Statement

IHAMS (NCT-01165463) was reviewed and approved by the University of Iowa (IRB Protocol 200908789), and funded by the NIH (RC1 AG035546). Written informed consent was obtained from all participants at baseline, and verbal consent was obtained at all follow-up interviews.

### Protocol and Adjustments

The protocol for this trial and supporting CONSORT checklist are available as supporting information (see Checklist S1 and Protocol S1). Five adjustments were made to the funded protocol prior to enrollment. First, direct randomization to four study arms within age bands was used, rather than initial randomization to three study arms with subsequent second level randomization to no booster vs. booster training within the on-site visual speed of processing training arm. Second, UFOV was specified as the sole primary outcome because no data existed from ACTIVE or any other study for the other neuropsychological outcomes, which were re-designated as secondary outcomes. Third, the power calculations were revised based on additional analyses of the one-year UFOV results from the ACTIVE study. Fourth, participants were trained in two-hour sessions, rather than the one-hour sessions used in ACTIVE due to the logistical and financial constraints of the funding mechanism. Finally, Blom rank transformations were used within age bands for normalization of the left and right censored neuropsychological outcomes, with linear mixed models used for consistency with prior studies.

### Design

**Figure 1:**
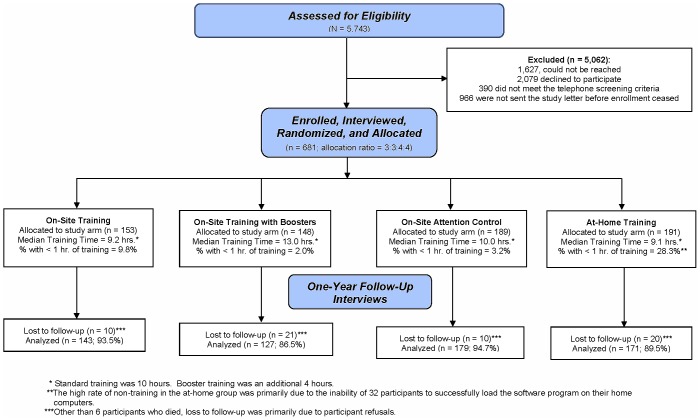
CONSORT Flow Chart for the IHAMS Study.

Details about the protocol [32] and post-training results on the primary outcome (UFOV) [33] have previously been reported. Figure 1 presents the IHAMS CONSORT flow diagram for the three visual speed of processing active intervention arms (commercially available as *Road Tour* from Posit Science Corporation, San Francisco, CA, USA) vs. the attention control computerized crossword puzzle game (commercially available as *Boatload of Crosswords*, Boatload Puzzles, Yorktown Heights, NY, USA).

### Sample

Sample size estimation was based on a secondary analysis of the ACTIVE data where the average one-year improvement in the UFOV score for the no contact controls was 69.9 ms (standard deviation = 159.1 ms) vs. 220.5 ms (standard deviation = 188.3) for the speed of processing group, for a 150.6 ms improvement differential. With 650 participants overall, a minimum of 138 in each group after 15% attrition by the one-year follow-up, and a reduced improvement differential of only 60 ms (due to IHAMS' inclusion of younger participants, use of an attention control group, and unconditional randomization to booster training), there would be 81.6% power (alpha = 0.05, two-tailed) for the primary outcome.

Potential participants were identified from patients attending the general internal or family medicine clinics at the University of Iowa. Using the electronic medical record (*Epic*, Verona, WI, USA), initial inclusion criteria were age (≥50), having≥2 visits to the clinic in the past year, and having no ICD9-CM codes indicating cognitive impairment. Cognitive impairment codes were Alzheimer's disease (331.0), Pick's disease (331.1), arteriosclerotic dementia (290.4 to 290.43), other senile or pre-senile dementia (290.0 to 290.9), dementia due to alcohol (291.1 to 291.2) or drugs (292.82 to 292.83), amnestic syndrome (294.0), or dementia due to other organic conditions (294.1). This electronic screening identified 5,743 potential participants.

Randomly selected (without replacement) weekly samples of 250 of these 5,743 individuals were then sent letters beginning in February 2010 and ending in November 2010 that were signed by the clinic medical directors and the principal investigator (FW) inviting them to participate by calling the IHAMS project office. Responding individuals underwent a brief screening telephone call to determine whether they met further inclusion criteria that could not be assessed using the electronic medical record. These were (a) having a PC and internet connection in the home, (b) no significant uncorrected self-reported vision issues, (c)<3 errors on the Short Portable Mental Status Screening Questionnaire [35], and (d) living within 30 miles of the project office. Of the 5,743 potential participants, 1,627 could not be reached, 2,079 did not respond or declined, 390 failed to meet one or more of the inclusion (telephone screening) criteria, and 966 had not been sent their letters by the time study enrollment closed.

### Randomization

The 681 eligible participants were invited to the project laboratory where written informed consent was obtained prior to their enrollment and baseline assessments, which were conducted by trained interviewers. Participants were then randomized by the study statistician (MJ) within age bands (50–64 and≥65) using a computerized algorithm, a 3∶3∶4∶4 allocation ratio, random permuted blocks of 4, 8, and 12, and sequentially numbered opaque envelopes [32], [33]. Because the participants' group assignments remained sealed until after the end of their initial visits, when the envelope was opened by the study coordinator (MD), the baseline assessments were fully double-blinded. After that, however, participants were no longer blinded because they knew the group to which they had been assigned.

### Training

After their baseline assessments and randomization, participants were invited back to the project laboratory on a subsequent day for their first training session. At that first session each on-site training participant received a scripted 15-minute introduction on how to use the assigned training program before going to one of two identical training rooms (one had the visual speed of processing program on five private work stations, while the other had the attention control program on five private work stations) to complete the first of their five weekly, two-hour training sessions. After completing 10 hours of training (the same basic training dose used in ACTIVE), or at approximately 6–8 weeks post-randomization for non-adherents (whichever came first), all on-site participants were invited back to the project laboratory for post-training assessments. One of the on-site active intervention training groups was also invited back at 11 months for two additional 2-hour “booster” training sessions (as in ACTIVE). All on-site participants were invited back for one-year assessments at which blinding was broken for the assessors but not for the investigative team at the concluding set of questions that solicited the participants' views about their assigned training program. After completing the one-year assessments, all on-site participants were given a copy of the visual speed of processing training software and installation directions to take home and load on their PCs and use at their discretion in perpetuity.

The at-home training participants were also invited back to the research lab after their baseline assessments, and received the scripted 15-minute introduction on how to use the visual speed of processing training program. This was followed by a 5–10 minute scripted introduction on how to load the training software onto their home PC. These participants were then given a copy of the training software and the loading instructions and asked to go home, load it on their PCs, and then complete 10 hours of training. Like the patients in the other three training groups, the at-home training participants were invited back for their post-training assessments at 6–8 weeks post-randomization, and for their one-year assessments. After completing the one-year assessments, at-home participants were told that they could continue to use the visual speed of processing training software on their home PCs at their discretion in perpetuity.

### Visual Speed of Processing Training

**Figure 2:**
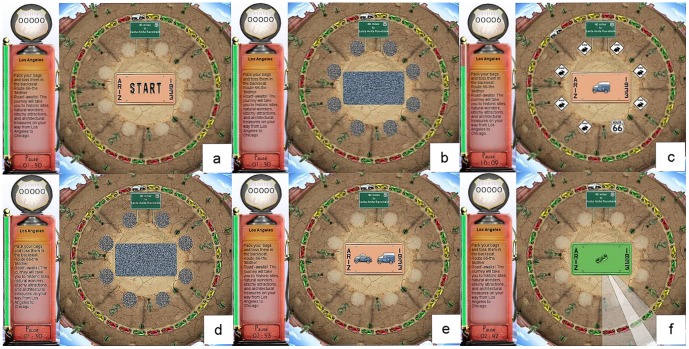
How the Visual Speed of Processing Training Program Appears to the User.

Figure 2 shows the appearance of the training program. After clicking on the start button in Figure 2a, Figure 2b is shown where the license plate and the eight circular locations surrounding it are masked. The masked license plate is then replaced (Figure 2c) with the target vehicle, either a car or a truck, and the eight masked circular locations are replaced with seven distracter stimuli (rabbit crossing signs in this example) or the target stimulus (always the Route 66 sign). The stimuli signs are presented for a specified time and are then replaced by Figure 2d. The amount of time that Figure 2c remains on the screen is measured in milliseconds (ms). In Figure 2e, both target vehicles (the car and truck) and the 8 circular locations are presented and the user is first required to select the correct target vehicle before selecting the circular location where the target sign appeared (Figure 2f).

Cognitive processing speed is trained by progressively reducing the ms of exposure that Figure 2c must remain on the screen for correct identification of both stimuli. The training program does not advance to a greater challenge until participants can maintain a 75% success rate at the current challenge level. As performance improves the challenge level is increased as follows: the visual field expands to add medium and distal orbits, these are accompanied by an increasing number of distracters (up to 47), and the vehicle pairs morph through nine different stages to become more similar and thus increasingly difficult to differentiate.

### Attention Control

The attention control group used a computerized crossword puzzle game for training purposes. This game offered a choice between three puzzle sizes, three levels of complexity, and varying font sizes. It also included optional help features such as filling in an unknown letter or word. There is, however, no progressive challenge to the user by increased speed, visual field size, number of distractors, or degree of difficulty of target stimulus differentiation. Thus, the crossword puzzle program provides an appropriate, computerized attention control alternative to the active visual speed of processing training.

### Outcomes

The primary outcome [33] was the UFOV [19], which was obtained at the baseline, post-training, and one-year assessments. It includes stimulus identification, divided attention, and selective attention subtests scored from 17-500 ms reflecting the shortest exposure time at which the participant could correctly perform each subtest, with a composite score ranging from 51–1500 ms. The secondary outcomes [33] were obtained at the baseline and one-year assessments. They were the Trail Making A and B tests (Trails [36]), the Symbol Digit Modalities Test (SDMT [37]), the Stroop Color and Word Test (Stroop [38]), the Controlled Oral Word Association Test (COWAT [39]), and the Digit Vigilance Test (DVT [40]). The Trails assess visual scanning ability, processing speed, and set-shifting/executive functioning, and are coded as the number of seconds to correctly complete connecting the number (Trails A) and number-letter (Trails B) sets. The SDMT is a substitution task that captures divided attention and processing speed, and is based on how many of 110 possible digit-symbol pairs are correctly scored in 90 seconds. The Stroop assesses the effect of interference with and inhibition on cognitive flexibility and processing speed, and other aspects of executive functioning, and is scored as the correct number of words, colors, and color-words identified in 45 seconds on each subtest. The COWAT is a phonemic test that assesses verbal fluency based on the number of unique words beginning with the letter C (or F or L) generated by the participant during 60 seconds, with a composite score of the number of correct words across the three letter trials. The DVT is a visual cancellation task that assesses sustained attention and psychomotor speed, and is performed by crossing out randomly placed 6′s in 59 rows of numbers, and is scored as the error (commission and omission) and time totals.

### Analyses

Means on the selected background characteristics and the neuropsychological outcomes were compared by intervention group and age band using chi-squared, analysis of variance, or Kruskal-Wallis tests for independent samples, as appropriate. The distributions on the neuropsychological tests are typically not normal due in part to left and right censoring (i.e., fixed minimal and maximal scores), and this was the case in IHAMS. At baseline, four of the outcomes were positively skewed (skewness = 1.3 to 3.0), six outcomes were leptokurtic (kurtosis = 1.3 to 7.1), and both distributional violations occurred for four outcomes (UFOV, Trails A, Trails B, and DVT errors). Therefore, Blom rank transformations were used to improve distributional normalization for model estimation, which resulted in means of zero and standard deviations of one [41], and yielded results that are directly interpretable as standardized effect sizes (Cohen's *d* [42]) because the effect of each intervention group vs. the attention control group is now measured in standard deviations. The Blom rank transformations were calculated separately within age bands to adjust for the different initial ability levels and to facilitate relative effect size comparisons.

Although attrition was modest (9%), it was evaluated using multivariable logistic regression (with backwards elimination given the small number of attriters) on the baseline neuropsychological tests, the treatment groups, age bands, and self-reported health [43]. Intent-to-treat analyses among the 620 (91%) respondents with complete data at the baseline and one-year assessments were conducted using linear mixed models. These models included between-person main effects for treatment group and age band, within-person main effects for time, and all two- and three-way interaction terms. Dunnett tests were used to compare each visual speed of processing training group to the attention control group. Significant observed effects were then converted into years of protection against age-related declines and/or years of improvement in cognitive performance by re-estimating the models using the original scalar values (i.e., non-Blom rank transformed scores), and dividing those effects by annualized age declines determined from prior normative studies. To address non-adherence to training (and provide a preliminary effectiveness analysis), especially within the at-home training group, the analyses were repeated after restricting the analytic sample to those who completed at least one hour of training.

## Results

### Attrition

91% of the participants completed their one-year assessments. To gauge the extent of differential attrition, the baseline neuropsychological tests, treatment groups, age bands, and self-reported health were all considered in an initial multiple logistic regression model. Only three of these variables were retained on the final step of the backwards elimination process. Participants in the booster training group had greater odds of being lost to follow-up (*adjusted odds ratio* [*AOR*] = 2.051, *p* = 0.021; the adjustment was for self-rated health and errors on the DVT test), as did those with lower self-reported health (*AOR* 1.597 per lower category rating, *p* = 0.004; the adjustment was for the booster training group status and errors on the DVT test), and those with more errors on the DVT test (*AOR* = 1.040 per error, *p* = 0.006; the adjustment was for booster training group status and self-rated health). Overall, the model fit the data reasonably well, with a *p* value of 0.710 on the Hosmer-Lemeshow statistic, and an area under the curve (*AUC*) of 0.690.

### Descriptive Statistics

Median training time was 13 hours for the on-site visual speed of processing training with boosters group vs. 9–10 hours for the other training and attention control groups. These average training times, however, mask substantial heterogeneity in adherence rates. Among those in the at-home visual speed of processing training group, 28.3% completed less than one hour of training vs. 9.8%, 2.0%, and 3.2% in the on-site visual speed of processing without booster training, on-site visual speed of processing with booster training, and on-site attention control groups, respectively (*p*<0.001). A substantial amount of the non-adherence in the at-home training group was due to 32 participants' self-reported inability to successfully load the visual speed of processing software on their home PCs, a problem that would likely be resolved using the web-based delivery method that is now commercially available.

Table 1 contains the group means for the background characteristics of the 620 participants in the analytic sample at baseline, by training group and age band with *p* values obtained from analysis of variance or chi-squared tests as appropriate. The only significant difference (*p*<0.05) involved marital status for the middle aged (i.e., 50–64 year old) adults, with the attention control group being less likely to be married. This difference was also the only one observed when the age bands were combined (data not shown). Table 2 contains the group means (prior to Blom rank transformations) for the neuropsychological tests at baseline by training group and age band with *p* values obtained using Kruskal-Wallis tests. Significant differences (*p*<0.05) were observed only for the SDMT for the middle aged adults, with the attention control group being somewhat disadvantaged (i.e., fewer correct pairs). When the age bands were combined (data not shown), however, significant differences (*p*<0.05) were observed for the SDMT, Trails B, and Stroop Word outcomes, with the attention control group again being somewhat disadvantaged (i.e., fewer correct pairs, slower times, and fewer correct words).

**Table 1:** Baseline Group Means on Background Characteristics by Treatment Group within Age Bands, among Participants with Complete Data at Baseline and One Year.

Variables	All Training Groups	On-Site VSP* Training	On-Site VSP* Training with Booster	On-Site Attention Control	At-Home VSP* Training		*p* value
Among Participants in the 50-64 Year Old Age Band							
Number of Participants	413	98	83	121	111		
Age (years)	57.2	57.9	56.8	57.0	57.2	0.267	
Sex (% women)	68.6	66.3	69.9	62.8	68.5	0.715	
Race (% whites)	94.2	92.9	92.8	94.2	96.4	0.542	
Married (%)	71.3	64.9	71.1	74.6	73.6	0.215	
Education						0.860	
(% with some college)	21.3	21.4	20.5	21.2	21.8		
(% college graduates)	71.9	73.5	73.5	71.2	70.0		
Employment						0.870	
(% working)	62.9	60.4	62.7	61.0	67.3		
(% retired)	17.2	19.8	15.7	16.9	16.4		
Self-Rated Health							
(% excellent or very good)	20.8	17.5	28.0	18.3	21.1	0.365	
Among Participants in the≥65 Year Old Age Band							
Number of Participants	207	45	44	58	60		
Age (years)	71.4	72.1	71.0	72.0	70.7	0.466	
Sex (% women)	54.6	68.9	52.3	43.1	56.7	0.072	
Race (% whites)	97.1	93.3	97.7	98.3	98.3	0.214	
Married (%)	74.0	59.1	75.0	82.8	75.9	0.037	
Education						0.207	
(% with some college)	21.0	18.2	15.9	19.0	28.8		
(% college graduates)	70.7	75.0	75.0	74.1	61.0		
Employment						0.651	
(% working)	17.6	20.5	15.9	12.1	22.4		
(% retired)	74.5	72.7	75.0	79.3	70.7		
Self-Rated Health							
(% excellent or very good)	21.5	22.7	18.2	24.1	20.3	0.942	

* VSP = visual speed of processing.

**Table 2:** Baseline Group Means prior to Blom Rank Transformations on the Primary and Secondary Outcomes by Treatment Group within Age Bands, among Participants with Complete Data at Baseline and One Year.

Variables	All Groups	On-site VSP* Training	On-Site VSP* Training with Booster	On-Site Attention Control	At-Home VSP* Training		*p* value
Among Participants in the 50–64 Year Old Age Band							
Number of Participants	413	98	83	121	111		
UFOV (ms)	247.2	224.4	231.4	273.1	250.9	0.265	
Trails A (sec)	39.3	37.4	38.6	42.2	38.4	0.178	
Trails B (sec)	60.3	58.7	58.0	64.5	59.0	0.175	
SDMT (# correct)	52.9	52.2	54.5	51.4	53.9	0.030	
DVT Errors (#)	7.2	7.2	7.1	7.6	7.0	0.630	
DVT Time (sec)	361.6	367.0	345.1	367.7	362.5	0.072	
COWAT (# words)	43.1	42.5	43.9	42.0	44.3	0.483	
Stroop Word (#)	72.5	73.3	74.1	70.3	73.1	0.131	
Stroop Color (#)	99.1	96.7	101.1	98.6	100.2	0.123	
Stroop Color-Word (#)	40.0	39.5	41.2	39.3	40.2	0.305	
Among Participants in the≥65 Year Old Age Band							
Number of Participants	207	45	44	58	60		
UFOV (ms)	386.5	407.0	384.1	415.8	343.5	0.170	
Trails A (sec)	46.1	43.1	46.5	49.1	45.0	0.286	
Trails B (sec)	77.9	77.7	70.7	86.3	75.1	0.082	
SDMT (# correct)	45.9	47.4	46.7	44.0	45.9	0.130	
DVT Errors (#)	10.1	11.7	9.5	9.4	9.8	0.834	
DVT Time (sec)	403.0	396.5	408.3	416.1	391.4	0.314	
COWAT (#)	39.6	41.1	39.2	38.1	40.1	0.443	
Stroop Word (#)	66.8	69.9	66.6	64.1	67.3	0.142	
Stroop Color (#)	95.4	97.3	97.5	91.0	96.8	0.072	
Stroop Color-Word (#)	34.9	35.6	34.4	33.7	35.8	0.613	

* VSP = visual speed of processing.

### Linear Mixed Models

Table 3 contains the results obtained from the linear mixed models of treatment group differences on the Blom rank transformed primary and secondary outcome measures. Cell entries are Cohen's *d* statistics (and associated Dunnett test *p* values) of the within-person changes in the neuropsychological outcomes from baseline to one year between each visual speed of processing training group vs. the attention control group. Cohen's *d* [42] statistics of 0.20 to 0.49 are considered small standardized effect sizes, Cohen's *d* statistics of 0.50 to 0.79 are considered medium standardized effect sizes, and Cohen's *d* statistics of 0.80 or greater are considered large standardized effect sizes. We note that there were no significant main effects for age bands, or for any interaction involving age bands. Thus, the standardized effect sizes shown were comparable for both age bands.

**Table 3:** Intent-to-Treat Linear Mixed Model Results for the Blom Rank Transformed Primary and Secondary Outcomes (the Comparator is always the Attention Control Group), N = 620.

	On-site Visual Speed of Processing Training	On-site Visual Speed of Processing Training with Boosters	At-Home Visual Speed of Processing Training
Primary Outcome			
UFOV Composite	−0.322** (−0.558, −0.089)	−0.579*** (−0.820, −0.338)	−0.372*** (−0.545, −0.149)
Secondary Outcomes			
Trails A	−0.261* (−0.500, −0.233)	−0.204 (−0.451, 0.043)	−0.265* (−0.493, −0.037)
Trails B	−0.225 (−0.463, 0.012)	−0.320** (−0.566, −0.074)	−0.263* (−0.490, −0.036)
SDMT	0.263* (0.014, 0.513)	0.351** (0.094, 0.609)	0.308** (0.070, 0.546)
Stroop Word	0.269* (0.019, 0.518)	0.271* (0.010, 0.531)	0.240* (0.001, 0.479)
Stroop Color	0.032 (−0.217, 0.282)	0.250 (−0.010, 0.510)	0.177 (−0.061, 0.416)
Stroop Color-Word	0.114 (−0.138, 0.366)	0.186 (−0.077, 0.450)	0.194 (−0.047, 0.435)
COWAT Composite	0.113 (−0.136, 0.362)	0.151 (−0.106, 0.409)	0.229 (−0.008, 0.466)
DVT Time	−0.061 (−0.313, 0.191)	−0.247 (−0.508, 0.014)	−0.205 (−0.444, 0.035)
DVT Errors	−0.111 (−0.345, 0.123)	−0.102 (−0.345, 0.141)	−0.049 (−0.272, 0.174)

* p<0.05; **p<0.01; ***p<0.001

Notes: Cell entries are Cohen's *d* statistics (95% confidence intervals) for visual speed of processing training on the baseline to one-year changes between each training group vs. the attention control group on the Blom rank transformed (separately within age strata) scores, and are directly interpretable as standardized effect sizes. Cell p values are from Dunnett test comparisons to the attention control group. None of the age band main effects were statistically significant, and none of the interaction terms involving age band were statistically significant.

**Figure 3:**
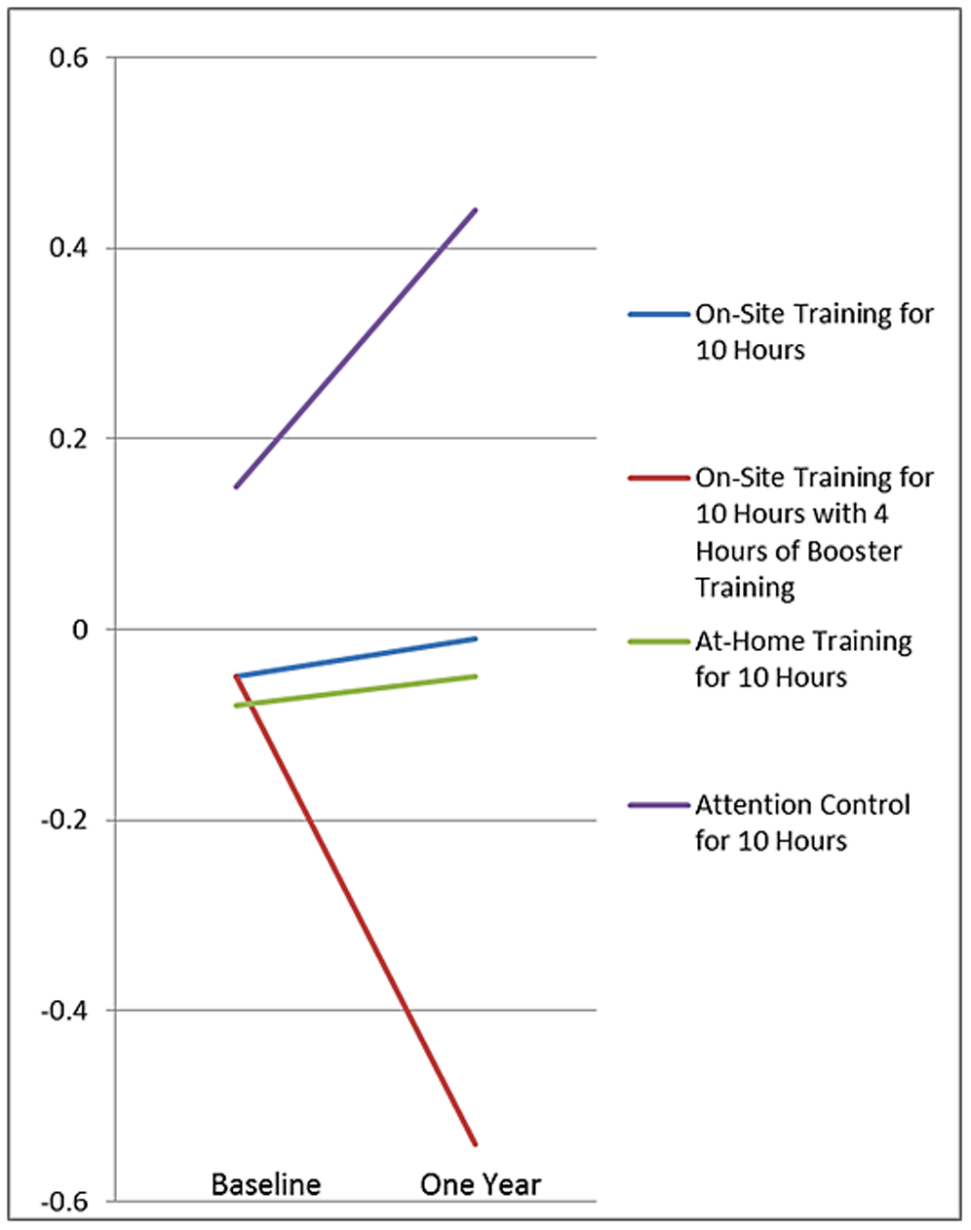
Plots of Blom Rank Transformed Means on the Useful Field of View by Treatment Group at Baseline and One Year.

Compared to the attention control group, all three visual speed of processing training groups had significant small to medium effect size improvements (i.e., faster completion times) on the UFOV (primary outcome). The difference between the differences observed for the on-site visual speed of processing with and without booster training (at 11 months) groups vs. the on-site attention control group reflects the effect of booster training. The differences observed for the on-site and at-home visual speed of processing training groups reflect the comparability of the effect of these two training delivery modalities despite the lower adherence rates in the at-home group. To better characterize these effect sizes, Figure 3 plots the Blom rank transformed means for the UFOV by treatment group at baseline and at the one-year follow-up. As shown, the attention control group experienced a one-year age-related decline in UFOV scores. In contrast, the on-site (with no boosters) and the at-home training groups were equally protected from that one-year age-related decline, while the on-site with booster training group had improved UFOV scores.

All three visual speed of processing training groups also had significant small effect size improvements (i.e., faster completion times, more correct symbol to digit matches, or more correct words) on four of the secondary outcomes—the Trails A, Trails B, SDMT, and Stroop word tests. In contrast to the effects on the UFOV, there was no additional benefit observed for booster training on any of the secondary outcomes. Consistent with the effects on the UFOV, visual speed of processing training was comparably effective when it was received in an unstructured at-home setting as it was in the structured project laboratory setting. To better characterize these effects, treatment group means at baseline and the one-year follow-up were plotted (data not shown). In general, these means plots revealed age-related declines for the attention control group, but protection against age-related declines for the treatment groups on the Trails A, Trails B, SDMT, and Stroop word tests. Although there were no statistically significant visual speed of processing training effects on the other secondary outcomes (Stroop color and color-word, COWAT, and DVT time and errors tests), all observed differences reflected larger improvements (more correct responses, faster times, and fewer errors) vs. the attention control group that were also comparable across delivery modes.

To gauge the clinical relevance of the significant intervention effects shown in Table 3, we converted them into years of protection against age-related declines and/or years of improvement in cognitive performance. This was done by re-estimating the models using the original scalar values (i.e., non-Blom rank transformed scores), and then dividing those effects by annualized age declines determined from prior normative studies. As shown in Table 4, the significant intervention effects reflect protection against normal age-related declines and/or improvements of 3.0 to 4.1 years (depending on intervention group) on UFOV, 2.2 to 3.5 years on Trails A, 1.5 to 2.0 years on Trails B, 5.4 to 6.6 years on SDMT, and 2.3 to 2.7 years on Stroop Word.

**Table 4:** Conversion of Standardized Effects^1^ into Years of Protection Against Age-Related Declines and/or Years of Improvement in Cognitive Performance (the Comparator is Always the Attention Control Group), N = 620.

	On-site Visual Speed of Processing Training	On-site Visual Speed of Processing Training with Boosters	At-Home Visual Speed of Processing Training
UFOV Composite^2^	3.0 years	4.1 years	3.2 years
Trails A^3^	3.5 years	2.2 years	3.0 years
Trails B^4^	1.5 years	2.0 years	1.8 years
SDMT^5^	5.4 years	6.6 years	5.9 years
Stroop Word^6^	2.7 years	2.5 years	2.3 years

1 Effects obtained from estimating the models shown in Table 3 using the original scalar values (i.e., non-Blom rank transformed scores). Those effects were then divided by the annualized age declines determined as documented in footnotes 2-6 below.

2 Annualized age declines of 15.6 ms were derived by using the Step 5 linear regression model from Edwards et al. [51] on the baseline UFOV composite (three subtests) among the no-contact controls in ACTIVE.

3 Annualized age declines of 1.26 seconds in Trails A were based on Table 2 in Tombaugh [52] for 65–69 vs. 70–74 year olds.

4 Annualized age declines of 3.82 seconds in Trails B were based on Table 2 in Tombaugh [52] for 65–69 vs. 70–74 year olds.

5 Annualized age declines of 0.41 correct symbol-digit pairs in SDMT were based on Table 9.8 in Lezak et al. [53] for 55–64 vs. 65–74 year olds.

6 Annualized age declines of 1.22 words in the Stroop Word test were based on the regression results for 60–87 year olds in Table 2 of Vogel et al. [54].

### Non-Adherence Sensitivity Analyses

Because of the significantly lower adherence rate in the at-home training group, the linear mixed model analyses were repeated after restricting the sample to those who completed at least one hour of training. With one exception those results were comparable to the analyses reported above. The exception involved the results for the primary outcome (UFOV). When non-adherent participants were excluded from the analyses the standardized effect sizes for visual speed of processing training were −0.278 for on-site training (*p*<0.05), −0.580 for on-site training with boosters at 11 months (*p*<0.001), and −0.524 for at-home training (*p*<0.001). Thus, when non-adherence was taken into consideration the observed effect sizes for the on-site with booster and at-home training groups were comparable.

## Discussion

In IHAMS, we addressed the five main limitations of the multi-site ACTIVE study by conducting an RCT of three methods of delivering the newest version of the visual speed of processing training that does not require ongoing supervision and which can be given to participants to use on their home PCs. IHAMS randomized 681 participants to 10 hours of on-site training, 10 hours of on-site training plus four hours of booster training at 11 months, 10 hours of self-administered at-home training, or 10 hours of on-site attention control (crossword puzzle program use). Among the 620 participants (91%) re-assessed at one-year, all three methods of delivering the visual speed of processing training had statistically significant small to medium standardized effect size (Cohen's *d* [42]) improvements (i.e., faster completion times) on the primary outcome (UFOV), with the on-site booster training group having the largest improvements. The larger improvements in the on-site with booster training group were expected, and the magnitude of these effects was consistent with those observed in previous studies using the original version of the visual speed of processing training with group (i.e., non-tailored) delivery protocols, including the ACTIVE study [21], [23]–[25]. The clinical relevance of these effects is that they translate into 3.0 to 4.1 years, depending on intervention group, of protection against normal age-related declines and/or improvements in UFOV performance.

What sets IHAMS apart from ACTIVE is that it was designed to be the first RCT to determine whether visual speed of processing training would work equally well for older and middle aged adults, and whether the training would affect other important neuropsychological outcomes. We found no significant differences in standardized effect sizes between the middle (50–64) and older (≥65) age bands. Thus, it is possible that visual speed of processing training may be used to address cognitive decline at least across those life course stages for which age-related cognitive decline has been demonstrated using the large, prospective Whitehall II cohort [16]. Because substantial cross-sequential data exist suggesting that age-related cognitive decline actually begins as early as age 28 [7], [8], [13]-[15], additional RCTs of visual speed of processing on adults at substantially earlier life course stages appear warranted.

IHAMS also breaks new ground in terms of visual speed of processing's ability to affect other important neuropsychological outcomes. We found significant effects reflected in small standardized effect size (Cohen's *d* [42]) improvements (i.e., faster completion times, more correct symbol to digit matches, or more correct words) on four of the secondary outcomes—the Trails A, Trails B, SDMT, and Stroop word tests. The clinical relevance of these effects is that they translate into 2.2 to 3.5 years of protection against age-related declines and/or years of improvement in cognitive performance on Trails A, 1.5 to 2.0 years on Trails B, 5.4 to 6.6 years on SDMT, and 2.3 to 2.7 years on Stroop Word. Moreover, while the improvements on the Stroop color and color-word, COWAT, and DVT times and errors tests were not statistically significant, they were all in the expected direction. That said, it is especially important to note here that several of these neuropsychological tests are more direct and commonly used measures of executive function than the primary outcome—UFOV. Therefore, these effects on the secondary outcomes suggest the potential for having beneficial cascading effects of as little as 10 hours of visual speed of processing training in many domains of everyday life that are highly affected by executive function.

Visual speed of processing training operates by requiring peripheral information processing (the Route 66 sign) in the presence of distractors (the rabbit crossing signs) simultaneously with performing a centrally located primary attention task (identification of the car or truck in the license plate). In addition to their effects on other aspects of everyday life [44], these targeted skills have repeatedly been shown to be especially important for the operation of motor vehicles and the retrospective, concurrent, and prospective reduction of accidents and collisions [45]. Indeed, in a six-year prospective follow-up to participants in the ACTIVE study, motor vehicle collisions were reduced by 43% in person-mile analyses and by 48% in the person-time analyses for the visual speed of processing training group compared to the no-contact control group [46]. And as previously noted, visual speed of processing training in ACTIVE has also been shown to result in significant and substantial long-lasting improvements on a number of health and quality of life outcomes [26]-[32].

For several reasons, our results have important implications for clinical practice and public health. First, age-related cognitive decline is common, and processing speed has been shown to play an early and central role in the cascading process that leads to many cognitive limitations [47]-[50]. Second, having a PC in the home has become relatively common, and with minimal instruction at the time of a clinical or public health encounter, patients could be given the visual speed of processing training software to take home and load onto their PCs and then use it there in private and at convenient times. Third, although we observed some adherence issues in the at-home training group, this issue likely could be addressed either by (a) a brief weekly engagement reminder delivered by e-mail or telephone, both of which could be automated, and/or (b) shifting to the new web-based version of the visual processing speed training that would overcome the difficulties faced by some participants in successfully loading the software onto their home PCs. Therefore, given the substantial evidence that it improves processing speed on a number of standard neuropsychological tests that focus on executive function, visual speed of processing training would appear to be a worthy weapon for consideration in the armamentaria of both clinical and public health practitioners in their battle against the common enemy of age-related cognitive decline.

Nonetheless, further research is needed to address several questions. These are whether: (1) the addition of brief weekly engagement reminders and/or the use of the web-based platform remediates the adherence issues observed for at-home training, (2) the training is effective for younger age bands (i.e., 30–50 year olds); (3) the observed effects last (although results from ACTIVE suggest endurance up to five years); and, (4) the training results in morphologic improvements to specific neural and structural mechanisms detectable using functional magnetic resonance imaging?

Finally, IHAMS is not without limitations, three of which warrant mention here. First, its respondents were predominantly white (95%), married (72%), college educated (71%), and healthy (68% reported excellent or very good health). Therefore, replication with more diverse and socioeconomically and health disadvantaged samples is needed. We note, however, that the ACTIVE study had a large (N = 2,802), racially and ethnically diverse, and socioeconomically disadvantaged sample, and that the effects of visual speed of processing training in it were comparable across race, ethnicity, socioeconomic, and health characteristics [22]–[32].

Second, IHAMS participants were generally cognitively preserved. For example, among those 65 years old or older, the mean on the UFOV at baseline in IHAMS was 386 ms (standard deviation = 182), which is noticeably faster than the mean of 485 ms (standard deviation = 250) at baseline on the same three subtests in ACTIVE. And among those 50–64 years old in IHAMS, the mean on the UFOV at baseline was even faster at 247 ms (standard deviation = 149; note that there are no age-comparable data from ACTIVE). Thus, a legitimate question is whether these statistically significant improvements on standard neuropsychological tests that target executive function are relevant. At this time we can only point to the empirical evidence shown in Table 4 that 1.6 to 6.6 years of protection against age-related declines and/or years of improvement in cognitive performance were achieved, even among the cognitively preserved IHAMS participants. Further research on more cognitively challenged individuals is needed to determine if comparable or even greater effects of visual speed of processing training can be achieved.

Third, despite a well-developed and carefully executed randomization protocol, significant differences (*p*<0.05) were observed when the two age bands were combined for the SDMT, Trails B, and Stroop Word outcomes (see Table 2) at baseline between one or more of the intervention groups and the attention controls. For these significant differences, as well as for other differences that did not reach statistical significance, the attention control group was generally somewhat disadvantaged (i.e., fewer correct pairs, slower times, and fewer correct words). While this is unfortunate, we found no evidence that it led to arbitrary results. Indeed, in sensitivity analyses we re-estimated the linear mixed model within median splits on the UFOV (the primary outcome) at baseline. The standardized effect sizes (Cohen's *d* statistics [42]) were statistically significant in both halves, but were larger in the slower performing half of the median split (Cohen's *d* = −0.341, *p* = 0.003; −0.671, *p* = 0.001; and −0.318, *p* = 0.004 for the on-site, on-site with boosters, and at-home training groups) than in the faster performing half (Cohen's *d = *−0.272, *p* = 0.019; −0.464, *p* = 0.001; and −0.375, *p* = 0.001, respectively).

In conclusion, we note that IHAMS successfully achieved all of its objectives and resolved the five limitations in the ACTIVE study. IHAMS demonstrated that all three modes of delivering the visual speed of processing training intervention significantly improved the primary outcome by clinically relevant amounts. Moreover, all three intervention arms also significantly improved several secondary outcomes that tap executive function (suggesting that the intervention effect was not merely “training to the test”), also by clinically relevant amounts. Finally, we observed comparable standardized effect sizes for both the middle and older age participants, underscoring the return on investment of beginning visual speed of processing training in middle age.

## Supporting Information

Checklist S1 CONSORT Checklist for the IHAMS Study.
(PDF)

Protocol S1 The Original NIH-Funded IHAMS Protocol.
(PDF)
