# Supplementary material for: A Randomized Controlled Trial of Cognitive Training Using a Visual Speed of Processing Intervention in Middle Aged and Older Adults
Source: PLoS One. 2013 May 1;8(5):e61624. doi: 10.1371/journal.pone.0061624 (PMC3641082; doi:10.1371/journal.pone.0061624)
Supplement: Protocol S1 — The Original NIH-Funded IHAMS Protocol. (PDF) [file pone.0061624.s002.pdf]

APPLICATION FOR FEDERAL ASSISTANCE
# SF 424 (R&R)

**2. DATE SUBMITTED:** 04/15/2009
**Applicant Identifier:**
**3. DATE RECEIVED BY STATE:**
**State Application Identifier:**

**1. * TYPE OF SUBMISSION**
☐ Pre-application ☒ Application ☐ Changed/Corrected Application

**4. Federal Identifier:**

**5. APPLICANT INFORMATION**
* Organizational DUNS: 0627616710000
* Legal Name: University of Iowa
Department: Division:
* Street1: 2 Gilmore Hall
Street2:
* City: Iowa City County: Johnson
* State: IA: Iowa Province:
* Country: USA: UNITED STATES * ZIP / Postal Code: 52242

Person to be contacted on matters involving this application
Prefix: * First Name: Twila Middle Name:
* Last Name: Fisher Reighley Suffix:
* Phone Number: 319-335-2123 Fax Number: 319-335-2130
Email: nih@uiowa.edu

**6. * EMPLOYER IDENTIFICATION** *(EIN) or (TIN):* 1-426004813-A1

**7. * TYPE OF APPLICANT:** H: Public/State Controlled Institution of Higher Education
Other (Specify):
**Small Business Organization Type** ☐ Women Owned ☐ Socially and Economically Disadvantaged

**8. * TYPE OF APPLICATION:**
☒ New ☐ Resubmission
☐ Renewal ☐ Continuation ☐ Revision

If Revision, mark appropriate box(es).
☐ A. Increase Award ☐ B. Decrease Award ☐ C. Increase Duration ☐ D. Decrease Duration
☐ E. Other (specify):

* Is this application being submitted to other agencies? Yes ☐ No ☒ What other Agencies?

**9. * NAME OF FEDERAL AGENCY:** National Institutes of Health

**10. CATALOG OF FEDERAL DOMESTIC ASSISTANCE NUMBER:** 93.701
TITLE: Trans-NIH Recovery Act Research Support

**11. * DESCRIPTIVE TITLE OF APPLICANT'S PROJECT:**
RCT of Two Speed of Processing Modes to Prevent Cognitive Decline in Older Adults

**12. * AREAS AFFECTED BY PROJECT** *(cities, counties, states, etc.)*
N/A

**13. PROPOSED PROJECT:**
* Start Date: 09/30/2009 * Ending Date: 09/29/2011

**14. CONGRESSIONAL DISTRICTS OF:**
a. * Applicant: IA-002 b. * Project: IA-002

**15. PROJECT DIRECTOR/PRINCIPAL INVESTIGATOR CONTACT INFORMATION**
Prefix: * First Name: Fred Middle Name:
* Last Name: Wolinsky Suffix:
Position/Title: Professor
* Organization Name: University of Iowa
Department: Health and Management Policy Division: College of Public Health
* Street1: E205 General Hospital
Street2:
* City: Iowa City County: Johnson
* State: IA: Iowa Province:
* Country: USA: UNITED STATES * ZIP / Postal Code: 52242
* Phone Number: 319-384-5129 Fax Number:
* Email: fredric-wolinsky@uiowa.edu

OMB Number: 4040-0001
Expiration Date: 04/30/2008

**16. ESTIMATED PROJECT FUNDING**

| | |
|---|---|
| a. * Total Estimated Project Funding | 998,933.00 |
| b. * Total Federal & Non-Federal Funds | 998,933.00 |
| c. * Estimated Program Income | 0.00 |

**17. * IS APPLICATION SUBJECT TO REVIEW BY STATE EXECUTIVE ORDER 12372 PROCESS?**

a. YES ☐ THIS PREAPPLICATION/APPLICATION WAS MADE AVAILABLE TO THE STATE EXECUTIVE ORDER 12372 PROCESS FOR REVIEW ON:

DATE:

b. NO ☒ PROGRAM IS NOT COVERED BY E.O. 12372; OR

☐ PROGRAM HAS NOT BEEN SELECTED BY STATE FOR REVIEW

**18. By signing this application, I certify (1) to the statements contained in the list of certifications* and (2) that the statements herein are true, complete and accurate to the best of my knowledge. I also provide the required assurances * and agree to comply with any resulting terms if I accept an award. I am aware that any false, fictitious, or fraudulent statements or claims may subject me to criminal, civil, or administrative penalties. (U.S. Code, Title 18, Section 1001)**

☒ *** I agree**

**** The list of certifications and assurances, or an Internet site where you may obtain this list, is contained in the announcement or agency specific instructions.***

**19. Authorized Representative**

| | |
|---|---|
| Prefix: | Dr. |
| * First Name: | Jordan |
| Middle Name: | |
| * Last Name: | Cohen |
| Suffix: | PhD |
| * Position/Title: | Interim Vice President for Research |
| * Organization: | University of Iowa |
| Department: | Office of the VP for Research |
| Division: | |
| * Street1: | 2 Gilmore Hall |
| Street2: | |
| * City: | Iowa City |
| County: | Johnson |
| * State: | IA: Iowa |
| Province: | |
| * Country: | USA: UNITED STATES |
| * ZIP / Postal Code: | 52242 |
| * Phone Number: | 319-335-2123 |
| Fax Number: | 319-335-2130 |
| * Email: | nih@uiowa.edu |

| * Signature of Authorized Representative | * Date Signed |
|---|---|
| Jordan Cohen | 04/15/2009 |

**20. Pre-application** Add Attachment Delete Attachment View Attachment

**21. Attach an additional list of Project Congressional Districts if needed.**

Add Attachment Delete Attachment View Attachment

OMB Number: 4040-0001
Expiration Date: 04/30/2008

# 424 R&R and PHS-398 Specific
# Table Of Contents


| | Page Numbers |
|---|---|
| **SF 424 R&R Face Page** | 1 |
| **Table of Contents** | 3 |
| **Performance Sites** | 4 |
| **Research & Related Other Project Information** | 5 |
| **Project Summary/Abstract (Description)** | 6 |
| **Public Health Relevance Statement (Narrative attachment)** | 7 |
| **Research & Related Senior/Key Person** | 8 |
| **Biographical Sketches for each listed Senior/Key Person** | 11 |
| **Research & Related Budget - Year 1** | 22 |
| **Research & Related Budget - Year 2** | 25 |
| **Budget Justification** | 28 |
| **Research & Related Budget - Cumulative Budget** | 31 |
| **PHS 398 Specific Cover Page Supplement** | 32 |
| **PHS 398 Specific Research Plan** | 34 |
| **Specific Aims** | 35 |
| **Research Design & Methods** | 36 |
| **Human Subjects Sections** | 48 |
| **Protection of Human Subjects** | 48 |
| **Women &Minorities** | 54 |
| **Planned Enrollment Table** | 55 |
| **Children** | 56 |
| **Bibliography & References Cited** | 57 |
| **Letters of Support** | 58 |
| **Resource Sharing Plan** | 59 |
| **PHS 398 Checklist** | 60 |

# RESEARCH & RELATED Project/Performance Site Location(s)

**Project/Performance Site Primary Location**

Organization Name: University of Iowa

* Street1: Room 2, Gilmore Hall

Street2:

* City: Iowa City County: Johnson

* State: IA: Iowa Province:

* Country: USA: UNITED STATES * ZIP / Postal Code: 52242

**Project/Performance Site Location** 1

Organization Name:

* Street1:

Street2:

* City: County:

* State: Province:

* Country: USA: UNITED STATES * ZIP / Postal Code:

**Additional Location(s)** Add Attachment Delete Attachment View Attachment

OMB Number: 4040-0001
Expiration Date: 04/30/2008

Close Form | Print Page | About

# RESEARCH & RELATED Other Project Information

1. * Are Human Subjects Involved? [X] Yes [ ] No

1.a If YES to Human Subjects

Is the IRB review Pending? [X] Yes [ ] No

IRB Approval Date:

Exemption Number: [ ] 1 [ ] 2 [ ] 3 [ ] 4 [ ] 5 [ ] 6

Human Subject Assurance Number: 00003007

2. * Are Vertebrate Animals Used? [ ] Yes [X] No

2.a. If YES to Vertebrate Animals

Is the IACUC review Pending? [ ] Yes [ ] No

IACUC Approval Date:

Animal Welfare Assurance Number

3. * Is proprietary/privileged information included in the application? [ ] Yes [X] No

4.a. * Does this project have an actual or potential impact on the environment? [ ] Yes [X] No

4.b. If yes, please explain:

4.c. If this project has an actual or potential impact on the environment, has an exemption been authorized or an environmental assessment (EA) or environmental impact statement (EIS) been performed? [ ] Yes [ ] No

4.d. If yes, please explain:

5.a. * Does this project involve activities outside the U.S. or partnership with International Collaborators? [ ] Yes [X] No

5.b. If yes, identify countries:

5.c. Optional Explanation:

**6. * Project Summary/Abstract** Abstract1002290609.pdf — Add Attachment | Delete Attachment | View Attachment

**7. * Project Narrative** ProjectNarrative1002290627.pdf — Add Attachment | Delete Attachment | View Attachment

**8. Bibliography & References Cited** Bibliography1002290610.pdf — Add Attachment | Delete Attachment | View Attachment

**9. Facilities & Other Resources** — Add Attachment | Delete Attachment | View Attachment

**10. Equipment** — Add Attachment | Delete Attachment | View Attachment

**11. Other Attachments** Add Attachments | Delete Attachments | View Attachments [ ]

OMB Number: 4040-0001

Expiration Date: 04/30/2008

# RCT of Two Speed of Processing Modes to Prevent Cognitive Decline in Older Adults

This application addresses broad Challenge Area 05, Comparative Effectiveness Research, specific Challenge Topic AG-102, Prevention and Risk Factor Reduction Strategies for Disabilities. The NIA contact is Ms. Georgeanne Patmios, 301-496-3138, *patmiosg@nia.nih.gov*. Challenge Topic 05-AG-102 calls for randomized controlled trials (RCTs) to evaluate the comparative effectiveness of competing interventions or modes of intervention delivery. A prime target for 05-AG-102 is the prevention of cognitive disability that results in health outcomes including improved quality of life, decreased mortality, morbidity, and disease progression, reduced medical care costs, and improvements in selected social and behavioral dimensions.

The largest and most rigorous RCT ever conducted involving long-term follow-up was the Advanced Cognitive Training for Independent and Vital Elderly (ACTIVE) study. Although all three ACTIVE cognitive training interventions (memory, reasoning, and speed of processing) were effective at improving their targeted abilities, the speed of processing group had the largest gains, with 87% of participants showing reliable improvement. We have also shown clinically significant effects of ACTIVE's speed of processing intervention (but no effects for the memory or reasoning interventions) on a variety of health outcomes, including: (1) a 3% reduction ($p = .012$) in predicted medical expenses; (2) a 38% reduction in the risk of global decline in health-related quality of life (HRQoL; $p = .004$); (3) a 30% reduction in the risk of worsening depressive symptoms ($p = .012$); (4) a 38% reduction in the risk of the onset of suspected clinical depression ($p < .01$); (5) improvements in self-rated health that translate to a 0.8% absolute reduction in the five-year mortality rate and a 10% relative mortality reduction ($p < .05$); and, (6) a 64% greater likelihood ($p < .05$) of meaningful improvements in internal locus of control.

Despite the magnitude, diversity, and endurance of these effects of the speed of processing intervention, further research is needed before widespread dissemination is warranted for three reasons. First, ACTIVE relied on a no-contact control group rather than an attention control group, raising the potential for placebo effects. Second, although booster training was randomly offered to 60% of ACTIVE participants, it was offered conditional on completing $\geq$ 8 of the 10 baseline training sessions, confounding booster effects with adherence effects. Third, a new, value-added version of the speed of processing software is now available that can be used on almost any home computer, and could thus dramatically reduce delivery costs and facilitate individual dosing and ongoing booster maintenance, but there is no published evidence that the value-added version is as effective as the original.

Therefore, our **specific aims** are to overcome these limitations using an RCT with one-year follow-up that can be fully completed within the NIH Challenge Grant two-year period. We will randomize 900 participants aged 50 years old or older to three groups. Group ***G1*** (N=400) will receive the value-added speed of processing intervention in 10 onsite sessions as in ACTIVE, with further randomization to one half (***G1a***) not receiving booster sessions and one half (***G1b***) receiving onsite booster sessions at 11-months. Group ***G2*** (N=250) will be the attention control group and will receive 10 onsite sessions using a computerized cross-word puzzle program. Group ***G3*** (N=250) will be shown how to operate the value-added speed of processing software on site, and will then be sent home to use it as often as they wish on their own personal computer. Our primary outcome measure is speed of processing, and we will use several reliable and valid instruments to provide a multidimensional assessment, including the Useful Field of View Test, the Symbol Digit Modalities Test, the Trail Making Test, the Controlled Oral Word Association Test, the Digit Vigilance Test, and the Stroop Color and Word Test. We have seven hypotheses for these primary outcome measures which will be tested using residualized change score multiple linear regression models for continuous outcomes, multiple logistic regression models for binary (threshold change) outcomes, and Poisson or negative binomial regression models for count measures. We will also evaluate the effects on several secondary outcomes, including HRQoL, healthcare utilization, depressive symptoms, functional abilities, perceived stress, self-efficacy, and sense of control. Finally, we will conduct stratified analysis among participants aged 50-64 years old, and separately among those aged 65 years or older in order to determine whether the effect size of the speed of processing intervention varies by age group.

## Public Health Relevance Statement

Although some degree of gradual cognitive decline is nearly universal and a normal part of the aging process, previous research by our group has shown that age-related cognitive decline is amenable to intervention. Building on speed of processing theory, we propose to extend and expand the findings from the NIH-funded, multi-site Advanced Cognitive Training for Independent and Vital Elderly (ACTIVE) study by using a newly developed, value-added version of the speed of processing software that can be used on virtually any home computer without supervision. When shown to be at least as efficacious as the original, the value-added version of the speed of processing software will then be ready for widespread implementation among adults aged 50 years old or older to reduce and/or prevent the risk of disability driven by age-related cognitive decline.

Close Form | Print Page | About

OMB Number: 0925-0001

# PHS 398 Research Plan

## 1. Application Type:

From SF 424 (R&R) Cover Page and PHS398 Checklist. The responses provided on these pages, regarding the type of application being submitted, are repeated for your reference, as you attach the appropriate sections of the research plan.

*Type of Application:

- [x] New
- [ ] Resubmission
- [ ] Renewal
- [ ] Continuation
- [ ] Revision

## 2. Research Plan Attachments:

Please attach applicable sections of the research plan, below.

| | | | | |
|---|---|---|---|---|
| 1. Introduction to Application (for RESUBMISSION or REVISION only) | | Add Attachment | Delete Attachment | View Attachment |
| 2. Specific Aims | SpecificAims1002290619.pdf | Add Attachment | Delete Attachment | View Attachment |
| 3. Background and Significance | | Add Attachment | Delete Attachment | View Attachment |
| 4. Preliminary Studies / Progress Report | | Add Attachment | Delete Attachment | View Attachment |
| 5. Research Design and Methods | ResearchDesignMethods100229 | Add Attachment | Delete Attachment | View Attachment |
| 6. Inclusion Enrollment Report | | Add Attachment | Delete Attachment | View Attachment |
| 7. Progress Report Publication List | | Add Attachment | Delete Attachment | View Attachment |

Human Subjects Sections

Attachments 8-11 apply only when you have answered "yes" to the question "are human subjects involved" on the R&R Other Project Information Form. In this case, attachments 8-11 may be required, and you are encouraged to consult the Application guide instructions and/or the specific Funding Opportunity Announcement to determine which sections must be submitted with this application.

| | | | | |
|---|---|---|---|---|
| 8. Protection of Human Subjects | ProtectionOfHumanSubjects10 | Add Attachment | Delete Attachment | View Attachment |
| 9. Inclusion of Women and Minorities | InclusionOfWomenAndMinoriti | Add Attachment | Delete Attachment | View Attachment |
| 10. Targeted/Planned Enrollment | TargetPlannedEnrollmentTabl | Add Attachment | Delete Attachment | View Attachment |
| 11. Inclusion of Children | InclusionOfChildren10022906 | Add Attachment | Delete Attachment | View Attachment |

Other Research Plan Sections

| | | | | |
|---|---|---|---|---|
| 12. Vertebrate Animals | | Add Attachment | Delete Attachment | View Attachment |
| 13. Select Agent Research | | Add Attachment | Delete Attachment | View Attachment |
| 14. Multiple PI Leadership Plan | | Add Attachment | Delete Attachment | View Attachment |
| 15. Consortium/Contractual Arrangements | | Add Attachment | Delete Attachment | View Attachment |
| 16. Letters of Support | | Add Attachment | Delete Attachment | View Attachment |
| 17. Resource Sharing Plan(s) | | Add Attachment | Delete Attachment | View Attachment |

18. Appendix | Add Attachments | Remove Attachments | View Attachments

# RCT of Two Speed of Processing Modes to Prevent Cognitive Decline in Older Adults

## SPECIFIC AIMS

In September 2006, the National Academies of Science (NAS, 2006) convened an interdisciplinary *Workshop on Identifying New Interventions to Extend Disability Decline in Elderly Populations* (hereafter, *The Workshop*), sponsored by the National Institute on Aging (NIA). The goal was "to consider specific low-cost interventions drawing on the lessons of demography, public health, economics, community medicine, and other fields" (NAS, 2006:1). NIA stressed its need "to outline a strategic vision for interventions to be conducted over the next 5-10 years" (NAS, 2006:2). Citing previous NIA-funded randomized controlled trials (RCTs), like the Advanced Cognitive Training for Independent and Vital Elderly (ACTIVE) study, NIA emphasized that future research must use RCTs to "confirm cause and effect, as well as impact" (NAS, 2006:2), especially since promising findings from epidemiologic disability prevention studies have often not been borne out in subsequent RCTs. NIA charged *The Workshop* to consider interventions that show impacts in as little as one-year, last as long as 5-10 years, and result in improved quality of life, decreased mortality, morbidity, and disease progression, reduced medical care costs, and psychosocial improvements.

When *The Workshop* was held, only preliminary analyses of ACTIVE had been completed; those results were not promising, suggesting the interventions had only "a very narrow and specific effect [that] did not generalize to other domains" (NAS, 2006:40). However, we have recently shown clinically significant effects of ACTIVE's speed of processing intervention on many secondary or health outcomes relative to the no-contact control group, including: (1) a $244 per person-year (3%) reduction ($p = .012$) in predicted medical expenses at one-year post-baseline (Wolinsky et al., 2009a); (2) a 38% reduction in the risk of global decline in health-related quality of life (HRQoL) at two-years ($p = .004$) and a 25.6% reduction in the risk of global decline in HRQoL ($p < .038$) at five-years post-baseline (Wolinsky et al., 2006a, 2006b); (3) a 30% reduction in the risk of worsening depressive symptoms at both one-year ($p = .012$) and five-years ($p = .023$) post-baseline (Wolinsky et al., 2009b); (4) a 38% reduction in the risk of the onset of suspected clinical depression at one-year ($p < .01$) post baseline (Wolinsky et al., 2009c); (5) improvements in self-rated health at two-, three-, and five-years equal to half the difference between "excellent" and "very good" responses ($p$ values $< .05$), which translates to a 0.8% <u>absolute</u> reduction in the five-year mortality rate and a 10% <u>relative</u> mortality reduction (Wolinsky et al., 2009d); and finally, (6) a 64% greater likelihood ($p < .05$) of improvements in internal locus of control at five-years post-baseline (Wolinsky et al., 2009e).

**Despite the compelling magnitude, diversity, and endurance of these impacts of the ACTIVE speed of processing intervention, further research is needed before widespread dissemination is warranted for three reasons.** <u>First</u>, although ACTIVE was a well-conducted RCT, it relied on a no-contact control group rather than an attention control group. While this was not problematic for the proximal or trained cognitive function outcomes (because those effects already had been shown vs. attention control), it did preclude eliminating the potential for placebo effects, especially with regard to the speed of processing intervention on health outcomes. <u>Second</u>, although four hours of booster training was randomly offered to about 60% of the participants in all three intervention arms, it was offered conditional on completing $\geq$ 8 of the 10 baseline training sessions. Thus, booster effects in the original ACTIVE trial cannot be separated from adherence effects. The <u>third</u> reason is that a new, value-added version of the speed of processing software is now available that can be used on almost any home-based personal computer, dramatically reducing delivery costs and facilitating individual dosing and ongoing booster maintenance. At this time, however, there is no published evidence that the value-added version is as effective as the original.

**Therefore, our <u>specific aims</u> are to overcome these limitations using an RCT with one-year follow-up that can be fully completed within the *NIH Challenge Grant* two-year period.** We will randomize 900 participants aged 50 years old or older to three groups. Group 1 (N=400) will receive the value-added speed of processing intervention in 10 onsite sessions as in ACTIVE, with half of this group further randomized to participate in four onsite booster sessions at 11-months post-training. Group 2 (N=250) will be the attention control group and will receive 10 onsite sessions using a computerized cross-word puzzle program. Group 3 (N=250) will be shown how to operate the value-added speed of processing software on site, and will then be sent home to use it as often as they wish on their own personal computer.

# RCT of Two Speed of Processing Modes to Prevent Cognitive Decline in Older Adults

## RESEARCH AREA

This *Challenge Grant* is in response to **Challenge Area 05**, Comparative Effectiveness Research, **Challenge Topic AG-102**, Prevention and Risk Factor Reduction Strategies for Disabilities. The NIA contact is Ms. Georgeanne Patmios, 301-496-3138, *patmiosg@nia.nih.gov*. **Challenge Topic 05-AG-102 clearly calls for RCTs that evaluate the comparative effectiveness of competing interventions or competing modes of intervention delivery.** Specifically identified as a prime target for 05-AG-102 is the prevention of cognitive disability that results in health outcomes including improved quality of life, decreased mortality, morbidity, and disease progression, reduced medical care costs, and improvements in selected social and behavioral dimensions. Our specific aims are to overcome the design flaws and software limitations of the speed of processing intervention previously used in ACTIVE using an RCT with one-year follow-up that can be fully completed within the *Challenge Grant* two-year period. We will randomize 900 participants aged 50 years old or older to three groups. Group 1 (N=400) will receive the value-added speed of processing intervention in 10 onsite sessions as in ACTIVE, with half of this group further randomized to be invited to participate in four booster sessions at 11-months post-training. Group 2 (N=250) will be the attention control group and will receive 10 on site sessions using a computerized cross-word puzzle program. Group 3 (N=250) will be shown how to operate the value-added speed of processing software on site, and will then be sent home to use it as often as they wish on their own personal computer. Ms. Patmios specifically invited this application in a March 5, 2009 e-mail to the PI, Dr. Wolinsky.

## THE CHALLENGE AND POTENTIAL IMPACT

Age-Related Cognitive Decline. Some degree of gradual cognitive decline is recognized as a normal part of the aging process. This age-related cognitive decline appears to be nearly universal, even among healthy elderly populations. This decline is evident across several domains of cognitive function including memory, orientation, attention, abstract thinking, and perception. It is important to distinguish these normal age-related cognitive declines from more severe impairments associated with dementia and sub-clinical cognitive impairment. From this developmental perspective, age-related cognitive changes can best be viewed as a continuum ranging from normal aging to mild cognitive impairment to dementia.

As the brain and the visual system age, numerous changes occur from the periphery through the central nervous system (CNS) which contribute to deficits in visual perception and cognition. Deficits are particularly notable in visual tasks requiring high levels of temporal precision (visual speed of processing) and attention (tracking multiple objects). These perceptual and cognitive deficits are significant contributors to the declines that emerge in visual cognition and in visually guided activities of daily living (ADLs; e.g., driving, preparing meals, and managing medication). Declines in visual accuracy and speed of processing can be understood, in part, as the consequence of CNS changes involving brain plasticity. Alterations in aging peripheral sensory inputs as well as changes in the use conditions of the brain over the lifespan could, through normal brain plasticity and learning processes, drive maladaptive changes in the speed and accuracy of information processing in the brain and result in the down regulation of neuromodulatory systems crucial for learning and memory. Overall, these brain-plasticity-driven changes are likely to be an important contributor to the speed, memory, and cognitive deficits common in normal old age.

The fact that age-related cognitive decline is nearly universal does not mean, however, that the decline is not amenable to intervention. Just as plasticity processes can lead to degradations in brain functioning with age, such processes can also serve as the basis for strengthening cognitive abilities. This recognition has led to considerable interest in developing training programs to help mitigate age-related declines in cognitive functioning. Numerous studies have investigated the effects of cognitive training programs targeting specific abilities affected by the aging process including memory, attention control, spatial orientation, inductive reasoning, figural relations, and artistic expression. In an effort to address more globally age-related changes in cognitive functioning, other interventions have targeted multiple domains. Although results vary, many of the interventions tested in these studies have demonstrated at least short-term promise with regard to improving targeted cognitive abilities.

Speed of Processing Theory. One of the cognitive changes associated with the aging process that has received considerable attention in recent years is decline in processing speed. Accumulating evidence indicates that age-related deteriorations in cognitive abilities are attributable, at least in part, to declines in information processing speed. According to this theory, many of the impairments in cognitive functioning that occur with age are the result of a decrease in the speed with which certain processing operations are executed. A variety of cognitive processes including episodic and working memory, verbal fluency, and reasoning abilities have been found to be associated with information processing speed in older adults. Salthouse (1996) has hypothesized that declines in processing speed adversely affect cognition via two primary pathways, which he termed the limited time and the simultaneity mechanisms. The limited time mechanism refers to the restriction in the amount of time that is available to successfully accomplish a task which tends to occur when certain cognitive processes are completed too slowly. The simultaneity mechanism operates when slowed information processing promotes the loss of the products of early cognitive processing through decay or displacement by the time they are needed for later operations. Extensive evidence supports the speed of processing theory of age-related cognitive decline. Importantly, associations between various subjective and objective measures of health status and cognitive functioning have also been found to be related to processing speed to a greater degree than to other higher order cognitive processes, suggesting an important link between speed of processing and a wide variety of health outcomes (Rosnick, et al., 2004). Moreover, Salthouse (2009) has recently shown that these age-related declines in speed of processing begin to occur when people are in their 20s and 30s.

Given the strong association between processing speed, functional abilities, and health status, interventions that successfully enhance speed of processing have the potential to facilitate performance and well-being in older adults. Several studies have investigated the impact of cognitive interventions to improve visual processing speed among older adults. The most rigorously tested visual speed of processing intervention with the greatest amount of empirical support is the Useful Field of Vision (UFOV) training program developed by Ball and Roenker (Ball, et al., 2007; Edwards, et al., 2005). UFOV training was designed to improve the efficiency and accuracy of visual information processing and the ability to perform complex visual attention tasks. Users are trained to improve the speed and accuracy with which they identify and locate visual information using a divided attention format. Over time, the difficulty and complexity of each task is systematically increased as users successfully attain specified performance criteria. Manipulations used to increase difficulty include decreasing the duration of visual stimuli presentation, adding visual or auditory distracters, increasing similarity between target and distracter stimuli, and presenting visual targets over a broader spatial expanse. In all configurations, however, the basic tasks are the same—central discrimination and peripheral target location.

The NIH-Funded ACTIVE Study. Although many cognitive training interventions have been conducted in an attempt to limit the onset and progression of disability, these generally have been hampered by numerous methodological limitations including small sample sizes, lack of random assignment, omission of control groups, and short-term follow-ups (Jobe, et al., 2001). Surprisingly few large-scale RCTs have attempted to investigate long-term changes following cognitive training programs. By far the largest and most methodologically rigorous RCT to date involving long-term follow-up was the Advanced Cognitive Training for Independent and Vital Elderly (ACTIVE) study, on which PI Wolinsky is a Co-I. A detailed description of the ACTIVE study is available elsewhere (Jobe, et al., 2001). Simply put, ACTIVE hypothesized that each of three intervention arms (memory, reasoning, and speed of processing training) would have a direct effect on its targeted, trained outcome (referred to as proximal outcomes), and nonspecific effects on each of its non-targeted, untrained outcomes (via social contact or cognitive arousal mechanisms). It was hypothesized that the effects of the ACTIVE interventions on both the primary and secondary outcomes would be mediated through the targeted, trained (proximal) outcomes. Among the primary outcomes, the reasoning and memory interventions were expected to affect only everyday problem solving and ADLs and instrumental ADL (IADLs) functioning, whereas the speed of processing intervention was hypothesized to have more diverse effects, including ADL and IADL functioning, everyday speed, and driving habits. All three ACTIVE interventions were expected to affect the secondary outcomes, including HRQoL, depressive symptoms, mobility, health services use and expenditures.

ACTIVE participants were $\geq$ 65 years old and lived independently in the community. Each of the six sites developed its own recruitment strategy. From March 1998 through October 1999, 4,970 potential participants were identified. Of these, 905 (18%) were excluded due to poor vision, dependency in personal hygiene or dressing, cognitive impairment or dementia of the Alzheimer's type, recent ischemic strokes,

cancer or active cancer therapy-based reduced life expectancies, communication difficulties, short-term plans to move or other anticipated scheduling conflicts, or participation in prior cognitive training programs. Another 1,263 potential participants (25%) were unwilling to participate. The 2,802 remaining potential participants were screened, signed written IRB-approved informed consent, and were enrolled in ACTIVE.

All data collectors completed an intensive four-day training workshop at the Coordinating Center, had to pass uniform certification requirements, and were subject to continuous quality improvement evaluation and review. Baseline data were collected after enrollment, and each of the six sites used a computerized program to randomize participants to the four study groups. Randomization was done immediately prior to the initiation of treatment in order to minimize drop-out and potential imbalances between randomization and treatment. At follow-up, data collectors were blinded to treatment assignment, and re-assessed participants immediately after training, and at 1-, 2-, 3-, and 5-years post-baseline.

The memory, reasoning, and speed of processing interventions each involved 10 standardized sessions that shared nine key elements (e.g., practice, individual, and group components, fostering self-efficacy, and social interaction), and involved 1-hour intervention sessions. The 10 sessions were spread over six weeks, with an optimum group size of 3-4 participants per group. The first five intervention sessions focused on strategy instruction and practice exercises, while the last five provided additional practice. Both the laboratory-type and everyday activities used were well-specified in trainer manuals, and the intervention trainers underwent extensive training, certification, and continuous quality improvement evaluation and review, with no cross-training permitted. About one month prior to the first and third annual follow-ups, booster training was offered to a 60% random sample of cognitive intervention participants conditioned on their having completed at least 80% of the initial training sessions. These participants received up to four additional standardized sessions at each follow-up under equivalent circumstances.

Inductive reasoning was the focus of reasoning training, especially the ability to solve problems that required linear thinking, followed a serial pattern, and were manifest in executive functioning. Such problems included understanding the daily dosing pattern for prescription drugs or using a bus schedule to plan transportation needs. Verbal episodic memory was the focus of memory training, especially using multiple mnemonic strategies for remembering lists, sequences of items, text material, and main ideas and story details. Sample problems included grocery shopping lists and visualizing and associating items to be remembered, such as "to-do" lists. Visual search and the ability to identify and locate visual information quickly in a divided attention format was the focus of the computer-based speed training (i.e., UFOV training). This involved systematically reducing the duration of the target stimulus presentation, progressively increasing the divided visual attention difficulty level and field.

Results from ACTIVE: Effects on the Proximal & Primary Outcomes. Although all ACTIVE treatments were effective at improving their targeted abilities (proximal outcomes) at posttest, the speed of processing training group demonstrated the largest gains, with 87% of participants showing reliable improvement after the intervention. Results published in *JAMA* indicate that each intervention continued to show improvements in their targeted cognitive abilities relative to baseline at both two- and five-year follow-ups (see Ball, et al., 2002; Willis, et al., 2006). The greatest relative improvements in targeted abilities, however, were clearly associated with the speed of processing group, for whom the effect sizes at all time points were more than double those associated with the other interventions. Booster sessions led to further improvements in targeted cognitive abilities for the speed of processing and reasoning groups at one-, two-, and five-years post-baseline. With regard to the primary outcomes, the speed of processing intervention was associated with marginally significant improvements in IADL difficulty (effect size = 0.26; 99%CI: -0.002, 0.51) at five-years. Furthermore, booster speed of processing training was associated with significantly greater improvement on a composite measure of the primary outcomes involving the speed of interacting with real world stimuli (timed IADL) and a complex reaction time task (Road Sign Test) at both one- and five-years. Booster speed training was also associated with significantly greater improvements in ADL and IADL functioning at one-year, and marginally greater improvements ($p < .10$) at two-years.

Results from ACTIVE: Effects on the Secondary or Health Outcomes. We have recently shown statistically and clinically significant effects of ACTIVE's speed of processing intervention on many of the

secondary or health outcomes, relative to a no-contact control group, including: (1) a $244 per person-year (3%) reduction (*p* = .012) in predicted medical expenses at one-year post-baseline (Wolinsky et al., 2009a); (2) a 38% reduction in the risk of global decline in health-related quality of life (HRQoL) at two-years post-baseline (*p* = .004), and a 25.6% reduction in the risk of global decline in HRQoL (*p* < .038) at five-years post-baseline (Wolinsky et al., 2006a, 2006b); (3) a 30% reduction in the risk of worsening depressive symptoms at both one-year (*p* = .012) and five-years (*p* = .023) post-baseline (Wolinsky et al., 2009b); (4) a 38% reduction in the risk of the onset of suspected clinical depression at one-year (*p* < .01) post baseline (Wolinsky et al., 2009c); (5) improvements in self-rated health at two-, three-, and five-years equivalent to at least half of the difference between "excellent" and "very good" responses (*p* values < .05), which is known to be associated with a 0.8% absolute reduction in the five-year mortality rate, and a 10% relative mortality reduction (Wolinsky et al., 2009d); and, (6) a 64% greater likelihood (*p* < .05) of improvements in internal locus of control at five-years post-baseline (Wolinsky et al., 2009e).

The Three Serious Limitations of ACTIVE. Given the remarkably protective effects of the speed of processing intervention on the proximal, primary, and secondary or health outcomes in ACTIVE, it would seem reasonable at this point to recommend its widespread implementation. But that is not what we propose. Our reluctance to do so is based on two serious design flaws in ACTIVE, and the fact that the speed of processing intervention (i.e., the original UFOV program; Ball, et al., 2007; Edwards et al., 2005), was not user-friendly, or appropriate for widespread distribution and self-administration. Taken together, these three factors seriously limit internal and external validity of the original ACTIVE study.

The two design flaws in ACTIVE involved the control group and the booster sessions. With regard to the control group, ACTIVE used a no-contact control group. This was justifiable for the proximal and primary outcomes because each of the three cognitive interventions (i.e., memory, reasoning, and speed of processing training) used in ACTIVE had previously been demonstrated to be successful in head-to-head comparisons with appropriate attention-control groups. Thus, for the proximal and primary outcomes, there was no need to protect against a placebo effect. This is not the case, however, for the secondary or health outcomes, especially with respect to the speed of processing intervention which was procedural (while the memory and reasoning interventions were not) and involved a computer-based delivery system that allowed the participant to proceed at her/his own pace. As a result, the potential for placebo effects on the secondary or health outcomes is not only possible, it is also plausible for the speed of processing intervention, which is basically the only intervention shown to have clinically relevant effects on the health outcomes. Accordingly, we propose a one-year RCT that uses an attention-control group. Participants randomized to the attention-control group will spend equal time receiving onsite training in the use of a computerized cross-word puzzle program which should not affect speed of processing. Only a one-year follow-up in this new RCT is needed because all we need to do is demonstrate that the placebo threat associated with ACTIVE's reliance on a no-contact control group does not exist. That is, we already have some evidence suggesting that the mechanism that enables exposure to the speed of processing training to be so effective on health outcomes is the resulting increase in speed of processing (Wolinsky et al., 2009d).

The second ACTIVE design flaw involved the booster training. Although four hours of booster training was randomly offered to about 60% of the participants in all three intervention arms at 11- and 35-months post-baseline, it was offered conditional on completing ≥ 8 of the 10 baseline training sessions. Because of this, the booster effects cannot be separated from adherence effects in an intent-to-treat framework. Thus, we do not know whether up to 10 hours (no booster sessions) or up to 14 hours (one round of booster sessions) were needed to achieve the one-year successes on the secondary or health outcomes. Accordingly, we propose that the ACTIVE-like speed of processing training arm of our RCT be further randomized to two subgroups. Both would receive the same 10 hours of onsite instruction and use of speed of processing as was done in ACTIVE. One of the two subgroups, however, would also be invited to participate in four additional hours of onsite booster training at 11-months post-baseline, allowing us to resolve the booster-dosing issue in an intention-to-treat format.

As indicated above, the third reason the speed of processing training used in ACTIVE (i.e, the original UFOV) is not ready for widespread implementation is that a new, value-added version of the speed of processing software is now available that can be used on almost any home-based personal computer,

dramatically reducing delivery costs and facilitating individual dosing and ongoing booster maintenance. At this time, however, no published evidence demonstrates that the value-added version is as effective as the original. Obtaining such evidence is prudent prior to the wide-spread implementation of the new, value-added speed of processing software. Moreover, given the ability to deliver the new, value-added version of the speed of processing intervention on almost any home-based personal computer, we need to determine whether the ACTIVE onsite approach, which is cumbersome and does not facilitate self-maintenance and ongoing individualized dosing, is necessary. That is, can participants be instructed in how to use the value-added version onsite, including how to load it on their home computers, and then proceed to use it at home as often as they like. This option is especially attractive because the value-added version can automatically generate progress reports over the internet or a standard modem connection to a secure, centralized server. Accordingly, we propose having a third group in our RCT which will be shown how to operate the value-added speed of processing software onsite, and will then be sent home to use it as often as they like on their own personal computer. This provides a comparative effectiveness analysis of the onsite vs. at home delivery methods, which has considerable implications for wide-spread implementation.

## THE APPROACH

<u>The Value-Added Speed of Processing Intervention</u>

We will use the value-added version of this speed of processing training program that is now commercially available from Posit Science Corporation (San Francisco, CA). Posit Science acquired the rights to the original UFOV training program developed by Ball and Roenker (Ball et al., 1988; Edwards, et al., 2005) in November 2007. They retained the tasks used in previous efficacy trials, but modified the delivery platform to be user-friendly and easily self-administered. These modifications dramatically improve the ease with which the intervention can be disseminated and implemented in a variety of settings and contexts. Furthermore, the addition of certain game elements improves user engagement and likely enhances compliance. The basic appearance or landscape of the program as seen by the user is shown in Figure 1a. After clicking on the start button, Figure 1b is shown. This then switches to Figure 1c, in which the target vehicle (in this case a car [rather than a truck]) is presented in the center of the screen, and a target sign (Route 66) is presented in one of eight locations in the periphery along with seven distracter stimuli (rabbit crossing signs). These stimuli are presented briefly and then disappear, as in Figure 1d. In Figure 1e, two vehicles are presented in the center of the screen, one of which was the previously presented target. The user must click on the correct target vehicle (car or truck) and then click on the location where the correct peripheral target (Route 66 sign) appeared. When a trial (identification of the vehicle in the center and Route 66 sign in the periphery) is correct, the user can then decide where to place a car in the ring of cars (Figure 1f). This represents the gaming aspect. When placement of the car produces a sequence of three cars of the same color, these cars disappear, and the user's car moves around the ring. This added gaming element serves to increase the user's level of engagement in the exercise. Each lap around the ring gets the user's car closer to the next destination. There are three levels: Arizona (desert); Texas (farm land); and Chicago (urban). Note that in this initial (early) trial of the exercise, there are only seven distracters in fixed locations. As the user progresses, however, three changes occur, all of which further increase task difficulty: (a) the target visual field expands (as shown in Figures 2a-c); (b) an increasing number of distracters is added (up to 47; see Figures 3a-b); and, (c) the vehicle pairs morph through nine different stages or pairs to become more similar over time (as shown in Figure 4).

<u>Overview of the Proposed Study Design</u>

We propose to overcome the three internal and external validity limitations of the multi-site ACTIVE study using an RCT with one-year follow-up that can be fully completed within the *NIH Challenge Grant* two-year period. As shown in Figure 5, we will randomize 900 participants aged 50 years old or older to three groups. We are specifically lowering the minimum age of eligibility here because of Salthouse's (2009) recent and compelling evidence that among healthy, educated adults, evidence of age-related decline in several cognitive domains (including processing speed) exists as early as the 20s and 30s. All three groups will initially receive a 1-hour overview and exposure session to the software and mode of use to which they have been assigned. <u>Group 1</u> (***G1***) (N=400) will then receive the value-added speed of processing

Figures 1a-f.  The Initial Road Tour Sequence.

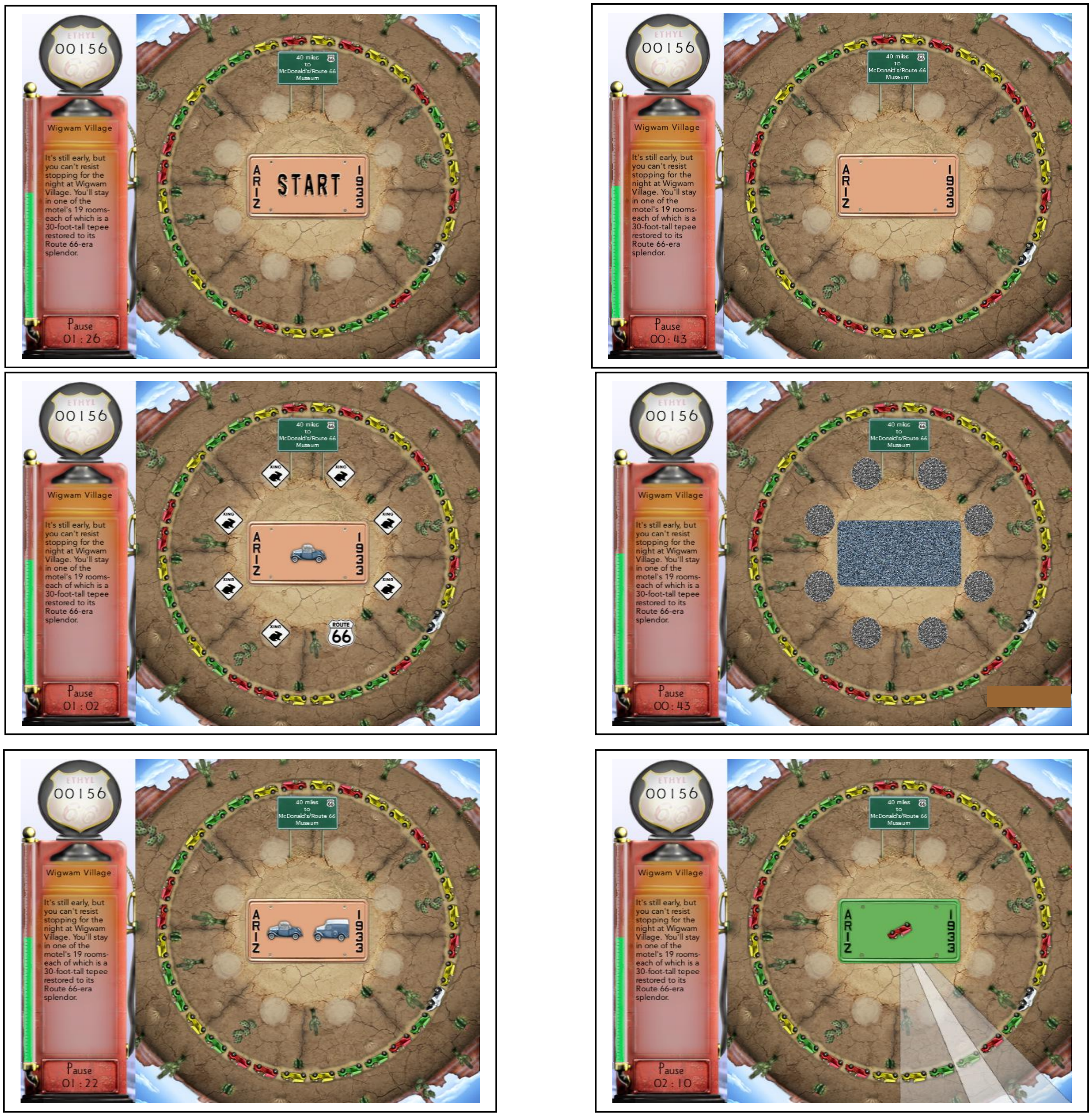


Figures 2a-c.  Expansion of the Target Visual Field.

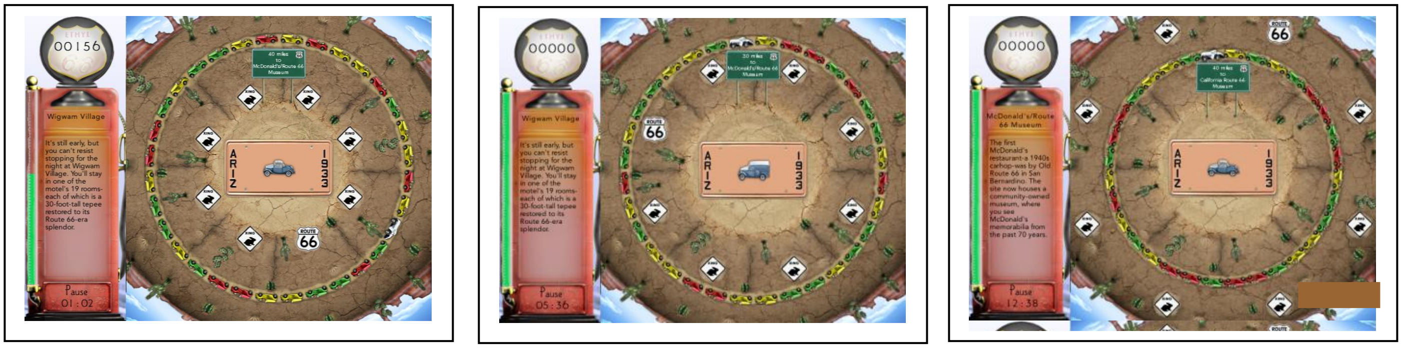

Figures 3a-b. Increasing the Number of Visual Distracters.

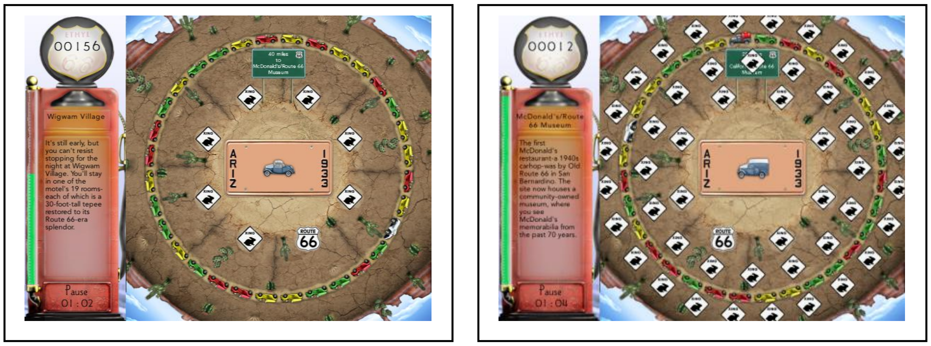

Figure 4. Car and Truck Morphing.

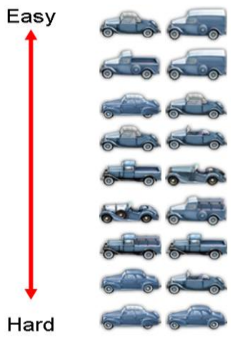


intervention delivered in 10 additional 1-hour sessions onsite as in ACTIVE, with half of this group randomized to not being invited to participate in four 1-hour booster sessions at 11-months post-training (***G1a***) and the other half of this group randomized to be invited to participate in booster sessions (***G1b***). Group 2 (N=250) (***G2***) will be the attention control group and will then receive an additional 10 1-hour on site sessions using a computerized cross-word puzzle program. After receiving the initial 1-hour exposure and orientation session to learn how to operate the value-added speed of processing software onsite, Group 3 (N=250) (***G3***) will then be sent home to use it as often as they wish on their own personal computer. Because of the potential value to older adults of the speed of processing training program, we are delighted to report that Posit Science has generously volunteered to provide the software (which is commercially priced at $395 per copy) without charge to the proposed study (please see the supporting letter in the Appendix). Because all 900 study participants will receive the software for their home use at the end of the study, this represents an in-kind contribution of $355,500 from Posit Science. Moreover, if shown to be at least as effective as the original UFOV software, Posit Science pledges to provide the value-added speed of processing training software at reduced prices for institutional or governmental purchase.

Figure 5. Study Design

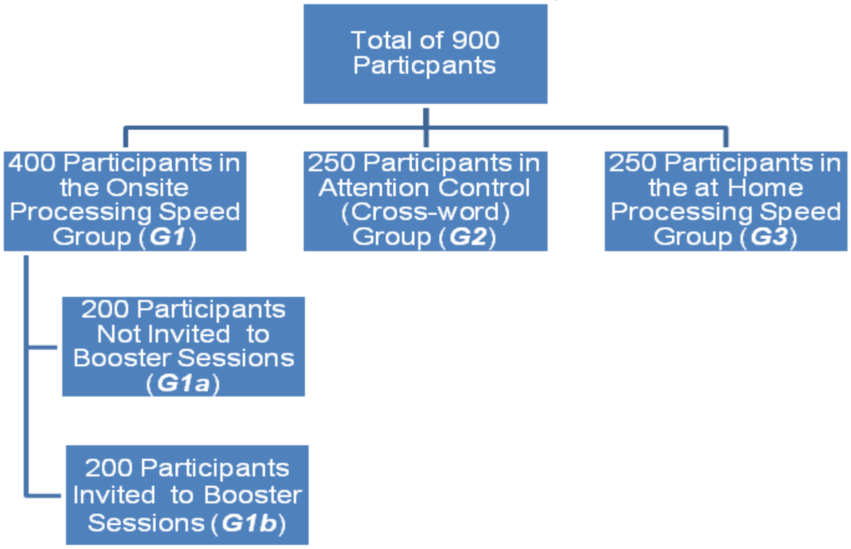


Participants, Random Assignment, and Retention and Attrition

We will recruit 900 participants, with minority participants oversampled to receive adequate coverage. We will recruit from the Family Practice (FP) and General Internal Medicine (GIM) Primary Care Clinics (PCCs) of the University of Iowa Hospitals and Clinics (UIHC). The electronic health records (EHR) of the PCCs at UIHC provide a fully automated system for selecting participants who meet our other inclusion criteria (after acquiring the necessary HIPAA waiver). The inclusion criteria include: (1) being at least 50 years of age, (2) receiving primary care from the PCC, and (3) being capable of providing informed consent. Although the Original UFOV training program has reliably demonstrated efficacy in improving

information processing speed and other outcomes among older adults ages 65+, Salthouse (2009) has recently demonstrated that age-related cognitive decline begins as early as the 20s and 30s. Therefore, half of our sample will be ages 50-64 in order to evaluate the effectiveness of the value-added speed of processing intervention in this younger age group, whereas the remaining participants will all be 65 or older, as in ACTIVE. This will enable us to investigate potential age-related differences in response to the interventions. To compare health care utilization and associated costs across the intervention conditions, we will include only individuals who have received care from the PCCs on at least two occasions in each of the preceding two years. This will ensure the inclusion of patients who receive care at the PCCs on at least a semi-regular basis. We will use block randomization to maintain balance on age across treatment conditions. Two strata representing the two levels of age (50-64 based on Salthouse's [2009] most recent work, and 65 and older as in the original ACTIVE study) will be created, within which participants will be block randomized to conditions. Block sizes will be varied and randomly determined from lengths of 3, 6, and 9 to maintain assignment concealment and to reduce the potential for selection bias.

The randomization procedures will include each of the following. First, the study biostatistician will determine the order of assignments using a computer-generated list of random numbers that is developed based on the randomization criteria described above. The assignment for each participant's ID number will be recorded on a participant letter and then sealed in an opaque envelope with only the ID number visible. Boxes containing the assignment envelopes will be stored in a locked cabinet in the Project Coordinator's office. The Project Coordinator will have the responsibility of unsealing the envelope and revealing each participant's group assignment. The following best practice procedures will be used to implement, control, and monitor the process. First, randomization procedures will be prepared, pilot tested, and revised as needed prior to enrolling participants. Second, randomization will be kept separate from the process of determining participant eligibility. Third, a single person (the Project Coordinator) will be responsible for revealing each participant's treatment assignment, with an additional staff person cross-trained to cover the process in the event of the Coordinator's absence. Fourth, the master list of assignments will be kept in a secure and locked location with access restricted to the Project Coordinator and study investigators. Fifth, the process will be carefully monitored throughout to ensure correct implementation, with a log used to document any protocol deviations. Finally, the Project Coordinator will meet regularly with the study investigators to review the process and to resolve any problems associated with the procedure.

Potential participants who meet any of the following exclusion criteria will not be eligible for enrollment: (1) presence of significant cognitive impairment, (2) lack of computer and Internet access in the home, (3) significant vision impairment, and (4) current possession or use of a Posit Science or other cognitive enhancement program. Because we are interested in the potential protective effects of the intervention against cognitive decline, and given that the intervention requires that users be able to attend to and follow directions independently, we will exclude patients who demonstrate evidence of significant cognitive impairment based on a diagnosis of dementia or a related cognitive disorder. Participants will also be asked to complete the Mini-Mental Status Examination (MMSE) at baseline as an additional screening measure, with those scoring ≤ 25 excluded. Note that although Internet access is needed to track home use of the processing speed intervention, it is not a general requirement for using the software at home.

FP and GIM PCC patients meeting initial age and use criteria will be identified from the UIHC EHR. Based on data from FY 2008, a total of 10,107 PCC patients meet the inclusion criteria for the study, including 4,044 (57.2% women) from FP and 6,063 (58.1% women) from GIM. On average, FP patients had 5.6 visits, and GIM patients had 5.9 visits to their PCC physicians, indicating that an adequate pool of potential participants who use the PCC as their regular source of health care exists. Patients will receive a letter describing the purpose of the study and inviting their participation two weeks prior to an upcoming primary care appointment. Patients will subsequently be approached at the time of their appointment about the study and their potential interest. Written informed consent will be obtained from those interested in participation, after which a brief screen will be conducted to determine eligibility. We have used a similar approach to successfully recruit participants for several of our previous clinical trials.

Eligible patients who opt to enroll in the study will be scheduled for a clinic visit for purposes of collecting baseline data. In order to reduce burden, participants will be given the option of completing the visit on the same day as their PCC appointment or coming in on a separate occasion. All primary and secondary outcome measures will be collected along with study covariates. Details regarding the condition to which the participant is assigned will be discussed, and he or she will be scheduled for their next study

visit to begin speed of processing training (or the Attention Control task). Based on our experience in ACTIVE, we expect attrition at one-year to be slightly less than 15%. For the current study, we anticipate even less attrition since our ongoing contacts with participants will be more frequent and because we have found in other research projects that PCC enrollees are generally less likely to drop out or withdraw from study participation. Moreover, we will make every effort to minimize attrition using state-of-the-art strategies including: (1) enrollment, consent, and baseline activities such as screening participants for availability and willingness to commit to long-term participation, fully informing participants of study requirements, gathering contact and tracking information, collecting names of personal contacts who can provide information about their whereabouts in the event they are lost to follow-up; (2) efforts to promote bonding with the study (e.g., sending newsletters and holiday cards); (3) maintaining high levels of contact; (4) hiring staff that are well-trained, enthusiastic, open, and responsive to participants; and (5) providing incentives to express appreciation and reinforce participation. The fact that participants' addresses and contact information will be maintained in the UIHC EHR in the event that they move will further facilitate a high rate of retention.

Data Collection, Entry, and Management

All data management activities will be centralized onsite in the UIHC. Standard procedures will be used for protocol development, preparations for data collection, data entry, data editing and database updating, reconciliation, database closure, data retrieval and statistical computing, and data security and confidentiality. Codes will be written so that missing and implausible values are automatically "flagged" for verification at the time data are entered. A double-entry procedure will be used so that all values will be entered on two separate occasions and compared for consistency. Discrepancies will be identified and corrected. Periodic reports and summary statistics will also be generated on a regular basis to examine for statistical outliers. All data will be downloaded into a master SAS database which will be appended daily. All data management procedures will be outlined in detail in the study Procedures Manual.

Screening will be performed at the initial visit. Eligible and consenting participants will receive their baseline assessment then, or be scheduled for baseline assessment at another time. All study measures (primary and secondary outcomes, and covariates; see below) will be administered at the baseline assessment, and re-administered at the one-year follow-up. After the baseline assessment, randomization to the three groups will occur, and participants will receive their 1-hour overview and exposure session to the software and mode of use to which they have been assigned. Participants assigned to Groups 1 and 2 (onsite speed of processing and onsite cross-word puzzle) will then be scheduled for their ten 1-hour onsite intervention sessions. At the end of the intervention training, all participants in Groups 1, 2, and 3 will receive post-tests using only the Useful Field of View Test. In contrast, participants assigned to Group 3 (at home speed of processing) will be given the software to take home and use after their 1-hour overview and exposure session. Participants in Group 3 will also receive weekly telephone calls for the first month to see if there were software installation problems, or other ongoing technical issues impinging on the use of the speed of processing program. At 11 months post-baseline, the half of Group 1 (onsite speed of processing) participants randomized to participate in four 1-hour onsite booster training sessions will be invited to receive them. They will be pre- and post-tested using the Useful Field of View Test.

Primary Outcome Measures

Because our primary focus is on demonstrating effects on various speed of processing measures, we have chosen several instruments to provide a comprehensive, multidimensional assessment of information processing speed. Each of the measures is brief, requiring approximately ≤ 5 minutes to complete. The first measure is the PC-administered mouse response version of the Useful Field of View Test, a stand-alone, four-task measure of visual information processing speed that taps: (1) stimulus identification only, (2) divided attention, (3) selective attention, and (4) selective attention combined with same/different discriminations. Scores represent the shortest display duration (in milliseconds) with which the respondent performs accurately on 75% of the trials. The Useful Field of View Test has demonstrably good test-retest reliability and concurrent validity when compared to the original Visual Attention Analyzer version of the test. Scores on the Useful Field of View Test are significantly associated with performance on IADLs and mobility as well as mental status, and are highly predictive of driving ability and accidents.

The Symbol Digit Modalities Test (SDMT) will be used to assess divided attention and processing speed. SDMT is a modified, inverse version of the Wechsler Digit Symbol test. In the SDMT participants view a key in which numbers (1-9) are paired with nine geometric symbols. Participants complete several practice items by writing the correct corresponding number in a space located below a geometric symbol. Participants are given 90 seconds to complete as many of 110 possible symbol-digit pairs as they can. Scores are based on the total number of correct pairings. The SDMT has good reliability and validity.

Visual scanning ability, processing speed, and set-shifting/executive functioning will be assessed using the Trail Making Test (TMT) Parts A and B. On Part A of the test, 25 numbered circles are distributed randomly over a sheet of paper. Participants are instructed to draw lines between the circles in ascending order from 1 to 25 as quickly as possible. For Part B, participants are asked to draw lines alternating between letters (A through L) and numbers (1 through 14) that are randomly arranged on a page. Scores are based on the time required to complete the test. The TMT is among the most widely-used neuropsychological tests and is considered to be one of the most sensitive measures of brain damage and progressive cognitive decline in dementia. The TMT has good ability to discriminate between patients with mild cognitive impairment, dementia, and healthy controls and measures executive function.

Verbal fluency will be assessed using the Controlled Oral Word Association Test (COWAT). Participants are given a letter and asked to generate as many words as they can that start with that letter in 60 seconds. Proper nouns, numbers and repetitions of the same word with a different suffix (e.g., catch and catching) are not allowed. A total of three trials are administered using the letters C, F, and L. The total number of allowable words that are generated for each trial serves as the score. The COWAT has good internal consistency and test-retest reliability.

Sustained attention and psychomotor speed will be assessed using the Digit Vigilance Test (DVT). The DVT is a number cancellation task in which participants are asked to find and cross out as quickly as possible specific target numbers (e.g., 6s) that appear randomly within 59 rows of single digits. Scores are based on the total time required to complete the task and the number of errors (omissions and commissions). The DVT has good reliability and discriminant validity.

Processing speed and executive functioning will be assessed with the Stroop Color and Word Test (SCWT), which consists of three separate trials involving three pages of stimuli. The first trial (Word naming) involves having participants name the words “red,” “green,” and “blue” that are repeatedly printed on a page in black ink. The second trial (Color naming) consists of having participants name the ink color with which a series of four X’s (*XXXX*) are printed. On the third trial (Color-Word), participants name the ink color when presented as non-matching color words (e.g., the word “red” printed in blue ink). Participants read as many colors or words as possible within 45 seconds. Whereas the Word and Color trials tap processing speed, the Color-Word task is a measure of executive functioning, as slowing occurs when the word and ink color are incongruent due to interference. The Stroop test has good reliability and validity.

Secondary Outcome Measures and Covariates

In addition to changes in processing speed, we are also interested in determining the impact of the speed of processing interventions on several secondary outcomes, including HRQoL, healthcare utilization, depressive symptoms, functional abilities, perceived stress, self-efficacy, and sense of control. For these we will rely on standard measures, many of which were used in ACTIVE, including the SF-36 for HRQoL, self-reports of healthcare use from the National Health Interview Survey, depressive symptoms using the CESD-12, functional abilities using the MDS ADL and IADL scales, the timed IADL, the Everyday Competency Test, sense of control (using the 2x2 Index), perceived stress (using the PSS-4), and self-efficacy (using the MacArthur battery). We will also collect information pertaining to sociodemographic and socioeconomic factors, existing medical conditions, and computer attitudes and experiences using standard measures at baseline. Changes in medical conditions will also be assessed at the 12-month follow-up.

Hypotheses and Statistical Analysis

As an initial step, descriptive statistics will be generated for all variables, stratified by intervention group and common time points (baseline and 12-month follow-up). For the purpose of modeling, we define the mutually exclusive 1-0 binary indicators ***G1*** and ***G3*** to indicate whether the participant is in the onsite

speed of processing intervention, or the at-home speed of processing group. The onsite cross-word puzzle (attention control; ***G2***) group subject has both indicators equal to zero. Other covariates are contained in the vector ***X*** (e.g., age, educational attainment, household income, etc.). For continuous outcomes like processing speed we will use residualized change score multiple linear regression (Kessler & Greenburg, 1981) to estimate a generalizeable equation that may be expressed in its simplest form as:

$$Y_{12} = \beta_o + \beta_1 Y_b + \beta_2 G1 + \beta_3 G3 + \beta_4 X + \varepsilon$$

where $Y_{12}$ is the dependent variable at the 12-month follow-up assessment, $\beta_o$ is the intercept, $\beta_1$ is the coefficient for $Y_b$ (which is the baseline value of the dependent variable), $\beta_2$ is the coefficient for ***G1*** (which is the binary indicator for the onsite speed of processing group), $\beta_3$ is the coefficient for ***G3*** (which is the binary indicator for the at home speed of processing group), $\beta_4$ is the coefficient for ***X*** (which is the vector of covariates), and $\varepsilon$ is the error term. In this equation, the effect of $\beta_1$ represents a stability coefficient, the effects of $\beta_2$ and $\beta_3$ represent the effects of the onsite and at home speed of processing interventions (respectively) on changes in the dependent variable compared to those observed for the reference (attention control) group, and the effects of $\beta_4$ represent the effects of the covariates on changes in the dependent variable. Note that for speed of processing there are six continuous outcome measures: Useful Field of View Test, SDMT, TMT, COWAT, DVT, and the SCWT. Although ACTIVE used a composite measure of processing speed, we plan to analyze performance on each measure separately in order to investigate more precisely the changes that occur in different components of processing speed. When the dependent variable is a count measure we will use Poisson or negative binomial regression techniques, and for binary outcomes (such as the onset of suspected clinical depression; CES-D 12 scores ≥ 9) we will use logistic regression (in which the value of $Y_b$ becomes 0).

As shown in Figure 5, the proposed design facilitates formal testing of seven key hypotheses (**Hn**) from an intent-to-treat perspective. Given the general equation shown above, **H1** involves the effect of $\beta_2$, which represents a replication of ACTIVE (i.e., onsite delivery) in which the basic <u>and</u> booster effects of speed of processing vs. the attention control group are pooled. **H1** hypothesizes that $\beta_2$ will be statistically significant and positive, indicating the benefit of being assigned to ***G1*** vs. the attention control group. To separate the basic effect (**H2**) from the basic plus booster effect (**H3**) among the onsite delivery group (***G1***) we substitute $\beta_{2a}G1_a$ and $\beta_{2b}G1_b$ for $\beta_2 G1$, where the ***a*** and ***b*** subscripts correspond to the subgroups randomized to basic, and basic plus booster. **H2** and **H3** hypothesize that both $\beta_{2a}$ and $\beta_{2b}$ will be statistically significant and positive, indicating the benefit of being assigned to either subgroup of ***G1*** vs. the attention control group. **H4** involves the effect of $\beta_3$, which represents the test of the at home delivery of speed of processing (***G3***) vs. the attention control group. **H4** hypothesizes that $\beta_3$ will be statistically significant and positive, indicating the benefit of being assigned to ***G3*** vs. the attention control group.

**H5** and **H6** allow us to evaluate the different modes of implementing the speed of processing intervention. **H5** contrasts $\beta_{2a}$ with $\beta_3$ and represents a head-to-head comparison of the basic onsite delivery vs. the at home delivery of the speed of processing intervention. **H5** hypothesizes that $\beta_3 > \beta_{2a}$ given the potential for individual dosing, maintenance, etc. in the at home delivery group. **H6** contrasts $\beta_{2b}$ with $\beta_3$ and represents a head-to-head comparison of the basic plus booster onsite delivery vs. the at home delivery of the speed of processing intervention. **H6** hypothesizes that $\beta_3 > \beta_{2b}$ given the potential for individual dosing, maintenance, etc. in the at home delivery group to exceed the four-session maximum in the onsite booster group. Finally, **H7** represents the head-to-head test of onsite basic or $G1_a$ to onsite basic plus booster training or $G1_b$. **H7** hypothesizes that $\beta_{2a} < \beta_{2b}$ with the difference reflecting the value of booster training. We do not specifically hypothesize any sex/gender-based or race/ethnicity-based differences, because no prior ACTIVE reports (Ball et al., 2002; Willis et al., 2006; Wolinsky et al., 2006a, 2006b, 2009a, 2009b, 2009c, 2009d, 2009e) have demonstrated any sex/gender-based or race/ethnicity-based differences in the effect of the speed of processing intervention on any proximal, primary, or secondary or health outcomes. Nonetheless, we will test for these effects in our analyses. We will also replicate all of our analyses within each age strata (50-64 vs. > 65) and compare effect sizes across strata.

In addition to the intent-to-treat testing of these seven hypotheses, we will also conduct effectiveness (as opposed to the above efficacy) analyses among those assigned to the at home speed of processing group or ***G3***. This will allow us to evaluate the effect of intervention dose based on the frequency with which the program is used at home. It will also enable us to address the issue of self-

selection to treatment frequency, since participants in this condition will not be randomly assigned to separate conditions representing the number of times they are exposed to the intervention. Specifically, using the tracking information automatically downloaded to the secure Posit Science server, we will sort ***G3*** participants into four groups based on their use of the speed of processing software. Those groups will include those who never used the software (which we hope will be too small for analytic purposes), and tertiles of use among those who used the software at least once. We will then develop a best fitting propensity score model using either multinomial or ordered logistic regression to predict group categorization using all available baseline assessment data. After adjusting for the propensity scores as in our prior work, we will calibrate return on investment dosing curves to identify an optimal dosing strategy.

Statistical Power

In generating effect size estimates, we based our calculations on results from the ACTIVE trial and a meta-analysis (Ball et al., 2007) which combined results from six prior clinical trials (with participants as young as 55) evaluating the original UFOV training, as well as adjustments that we expect based on the different implementation modes (onsite vs. at home). Consistent with ACTIVE and similar studies, we expect participants assigned to the attention control group to exhibit a slight worsening over time on measures of processing speed. Those assigned to the onsite speed of processing group are expected to demonstrate comparable treatment effects to those observed in the speed of processing group in the ACTIVE trial given the similar duration (10 weeks) and intervention delivery mode (onsite). We further hypothesize that participants in the at home speed of processing group will exhibit slightly greater protective effects due to the opportunity to self-dose and increase their overall level of exposure to the intervention.

We will have ≥ 80% power to detect differences between groups (including between subgroups randomized to be invited vs. not be invited to booster training in the onsite speed of processing group) as small as 75 ms on the Useful Field of View Test using a two-tailed comparison with $\alpha$ set at 0.05. This corresponds to an effect size of 0.32, which is small by standard criteria and is well within the median post-treatment effect size of 0.84 found in the meta-analysis of previous trials. It is also much smaller in magnitude than the effect size associated with comparisons between the speed of processing and no-contact control groups at the two-year follow-up in ACTIVE (effect size of 0.72).

### TIMELINE AND MILESTONES

We anticipate no changes in the course or direction of the continuing research effort for three reasons: (1) we propose a straightforward RCT with three groups—onsite delivery of speed of processing training (***G1***; further randomized into not being invited to booster sessions [***G1a***] or being invited to booster sessions [***G1b***]), an attention control group receiving an onsite cross-word puzzle intervention (***G2***), and an at home delivery of the speed of processing intervention (***G3***); (2) we use a value-added version of a previously validated and tested intervention; and (3) we have considerable experience in conducting RCTs and using this intervention. Nonetheless, at every critical juncture shown in the Gantt chart in Figure 6, we will evaluate whether changes in the course or direction of the research are warranted.

Figure 6. Study Gantt Chart

| **Study Tasks/Months** | 1 | 3 | 5 | 7 | 9 | 11 | 13 | 15 | 17 | 19 | 21 | 24 |
|---|---|---|---|---|---|---|---|---|---|---|---|---|
| Hire study staff and database preparation | ■ | | | | | | | | | | | |
| Secure IRB approval | ■ | | | | | | | | | | | |
| Staff training | ■ | ■ | | | | | | | | | | |
| Recruitment database | ■ | ■ | | | | | | | | | | |
| Baseline data collection | | ■ | ■ | ■ | ■ | ■ | | | | | | |
| Intervention delivery | | ■ | ■ | ■ | ■ | ■ | ■ | | | | | |
| Booster delivery | | | | | ■ | ■ | ■ | ■ | ■ | ■ | | |
| Follow-up data collection | | | | | | ■ | ■ | ■ | ■ | ■ | ■ | |
| Data analysis | | | | | | ■ | ■ | ■ | ■ | ■ | ■ | ■ |
| Manuscript preparation | | | | | | ■ | ■ | ■ | ■ | ■ | ■ | ■ |

# E. Human Subjects

## E.1. Risks to Subjects

### E.1.a. Human Subjects Involvement and Characteristics

The proposed study is a three-group randomized clinical trial designed to evaluate the effect of two modes of delivering a speed of processing training program (onsite vs. at home) to reduce or avoid cognitive decline vs. an attention control (cross-word puzzle) group. Participants will include 900 primary care patients being treated at the UIHC PCC, either in GIM or FP.

**Eligibility** criteria will include:

1) At least 50 years of age
2) Receive primary care from the UIHC PCC
3) Received care from the UIHC PCC on at least two occasions in each of the preceding two years
4) Be capable of providing informed consent

**Exclusionary** criteria will include:

1) Presence of significant cognitive impairment (based on a diagnosis of dementia or a related cognitive disorder and/or a Mini-Mental Status Examination [MMSE] score of ≤ 25 at the time of their baseline visit)
2) Lack of computer and Internet access in the home
3) Significant vision impairment (as determined by self-reported difficulty reading normal newspaper print [even with corrective lenses] and/or an Activities of Daily Vision Scale score of < 20)

No vulnerable populations will be included in this research project. Specifically, fetuses, neonates, pregnant women, children (under the age of 18), prisoners, institutionalized individuals, and other vulnerable populations will not be included.

### E.1.b. Sources of Materials

All data will be collected specifically for research purposes. These data will include contact information, self-report questionnaires, and several performance-related measures. Each of these sources of data is briefly described below.

E.1.b.1. <u>Contact Information</u>

Standard procedures will be used to track and retain study participants which include: (1) collecting names, addresses, and phone numbers of at least three relatives and/or friends who will always know each participant’s whereabouts; (2) contacting participants with personalized letters and cards; (3) maintaining phone contact with participants throughout the study; (4) setting up a (secure and access-restricted) tracking file with names, addresses, phone numbers, and projected visit windows; and (5) taking a case-management approach for approaching participants who have a difficult time attending study visits. All contact information will be stored on a secure file server and will be kept separate from study data.

E.1.b.2. <u>Self-report Data</u>

Participants will be asked to complete several self-report questionnaires to assess each of the following:

1) Socio-demographics (Items from Behavioral Risk Factor Surveillance System)
2) Health-related quality of life (HRQoL; SF-36)
3) Depressive symptoms (CESD-12)
4) Self-rated health (Single item from SF-36)
5) Health care utilization (Items from AHEAD study)
6) Activities of daily living (ADL; Items from Minimum Data Set – Home Care)
7) Instrumental activities of daily living (IADL; Items from Minimum Data Set – Home Care)

8) Self-efficacy (McArthur Studies of Successful Aging measure)
9) Sense of control (2 x 2 Index)
10) Computer experiences (Attitudes toward Computers Questionnaire [ATCQ])

E.1.b.3. Performance-related Measures

Several performance-related tests and measures will also be administered to assess domains related to aging and cognitive. These will include:

1) Useful Field of View Test (UFOV)
2) The Symbol Digit Modalities Test (SDMT)
3) Trail Making Test (TMT)
4) Controlled Oral Word Association Test (COWAT)
5) Digit Vigilance Test (DVT)
6) Stroop Color and Word Test (SCWT)

A brief description of each of the performance measures is provided in the methods section of the application.

**E.1.c. Potential Risks**

There is a small risk of loss of confidentiality associated with study participation. However, significant steps will be taken to protect participant confidentiality. Information collected during the project will be kept confidential within the limits allowed by law, and scientific publications will present data in such a way that it is not possible to determine the identity of individual participants. All computerized data will be kept on a secure server that is password protected, with access limited to study investigators, the project coordinator, data analyst, and data manager. All study staff will be trained in confidentiality procedures and will receive the required University of Iowa data security training.

The risks associated with the cognitive training interventions and study-related procedures are expected to be minimal. Some of the questionnaires assess potentially sensitive information, which could make some participants uncomfortable. Therefore, participants will be instructed that they can choose not to answer any questions if they so choose. Furthermore, some of our recent analyses (unpublished) using data from the ACTIVE study did indicate that participants who scored above a cut-off on a screen for possible clinical depression (defined as a score of ≥ 9 on the 12-item CESD-12) and who were assigned to the speed of processing intervention were less likely to “recover” (i.e., score below the cut-off) on subsequent annual assessments than those assigned to a control condition. It should be noted, however, that participants assigned to the speed of processing intervention who scored below the cut-off at baseline showed more favorable responses on depression outcomes at follow-up. Nevertheless, we will track participants for a significant worsening of depressive symptoms over time and recommend that participants who exhibit potentially significant levels of depressive symptoms see their primary care provider for additional evaluation and treatment as appropriate. Finally, there may be some risks associated with randomization such that one of the intervention conditions may prove to be more effective than the others at improving well-being and age-related outcomes. However, all participants will receive a copy of the speed of processing software program upon completing the study. Therefore, receipt of any benefits associated with exposure to either of the cognitive training programs will at most be delayed.

**E.2. Adequacy of Protection against Risk**

**E.2.a. Recruitment and Informed Consent**

Our primary recruitment strategy will be to identify participants through the PCC at the UIHC, specifically those attending the GIM and FP clinics. During the recruitment/enrollment phase, the participant will be informed not only of the details of the study but also of the fact that participation in this study is entirely voluntary and will not affect their current or future medical care at any UIHC facility. This will again be reinforced through the informed consent process. In addition, those who elect to participate will be clearly informed that they may withdraw from the study at any time without jeopardizing current or future care at the UIHC. The consent form will be reviewed with each participant and each participant will

receive a copy of the signed consent form. Standard language in our consent form assures the participants of the confidential nature of the study, in addition to allowing the participant to withdraw from the study at any time without adversely affecting current or future medical care at the UIHC. The consent forms will contain HIPAA language. These standards are strictly adhered to and monitored by the University of Iowa IRB.

**E.2.b. Protection against risk**

Several procedures will be in place to minimize participants' risks:

E.2.b.1. Voluntary Participation

It will be made clear to all participants that participation is voluntary and that they can withdraw from the study if their initial or on-going experience makes it oppressive, burdensome, or otherwise uncomfortable. It also will be made clear that they are free not to participate in any portion of the program without prejudice.

E.2.b.2. Confidentiality

Information collected during the project will be kept confidential within the limits allowed by law, and scientific publications will present data in such a way that it is not possible to determine the identity of individual participants. To ensure participant confidentially no names, social security numbers, hospital or clinic numbers will be included in the shared databases. Names, addresses, telephone numbers, and any other information needed for recruitment, study involvement, and tracking will be obtained and maintained locally by project personnel. All data will be maintained on a secure file server or in locked research data storage rooms to which only designated project staff will have access. All computer files and systems will be password protected and accessible by authorized personnel only. Data entry and transfer will be performed by trained data entry systems staff. All data will be maintained using SAS datasets.

E.2.b.3. Human Subjects Review and Training

All key personnel have completed the required education on the protection of human research subjects provided by the University of Iowa Human Subjects Office. The University of Iowa requires that all personnel complete human subjects training before they can engage in a research study. The University of Iowa IRB keeps a database with the training records and ensures all Investigators are certified. The IRB offers a web-based training through the CITI Company, or a face-to face- training with IRB members. Both trainings provide a history of ethical guidelines in human subjects protections, how federal regulations have evolved, the composition and duties of an institutional review board (IRB), the requirements for informed consent, the different types of IRB review, and investigator responsibilities. The Iowa IRB also accepts the NIH online Human Participant Protection training.

E.2.b.4. Operations Manual

An operations manual will be developed for the study, and the procedures will be strictly followed. All study personnel will be thoroughly trained in all study procedures in which they will be involved including recruitment, informed consent, measurements, randomization, participant follow-up, assessing, documenting, and reporting adverse events, and outcome determination. Adherence to the procedures in the operations manual will be assured by periodic assessment and internal auditing. Retraining will be conducted on a semi-annual basis (or more frequently as needed).

E.2.b.5. Monitoring Adherence

Monitoring adherence is critical in a clinical trial, both for safety considerations and because interpenetration of study results must take into account whether the intervention regimen was followed. Great emphasis will be placed on developing a sophisticated system for monitoring, scheduling, and tracking study participants. Every detail of all of the study visits will be planned and staff roles clearly delineated. A detailed appointment reminder and telephone follow-up system will be implemented. All participants will be mailed visit reminders and receive phone calls on the day prior to scheduled visits. All

missed appointments will be followed up within 24 hours by a call to the participant or to someone on the list of contact that he or she has provided. If the appointment has been missed, another appointment will be made promptly. A concerted effort will be made to achieve all appointment windows. UIHC clinics have a sophisticated EHR database system for tracking all study participants. Appointment windows and protocol adherence status are tracked constantly. This database allows us to identify any individuals who are outside of their visit windows. To increase adherence and regular attendance, study personnel will maintain close contact with participants, make study visits pleasant and convenient, and provide clear written and verbal instructions regarding study procedures and appointments.

### E.3. Potential Benefits of the Proposed Research to the Subjects and Others

It is uncertain whether participants will benefit directly from being in this study. However, based on findings obtained using data from the ACTIVE trial, it is possible that participants may experience improvements in some visual cognitive abilities as well as benefits to other related areas (e.g., HRQoL) as a result of one of the two active study interventions. In addition, all participants, including those in the Attention Control condition, will receive a copy of the speed of processing computer software at the end of the study free-of-charge for their own use after all study measurements have been completed. We also hope that, in the future, other people might benefit from this study through what we learn about the relative effectiveness of various cognitive training programs at reducing age-related cognitive decline.

### E.4. Importance of Knowledge to be Gained

Age-related cognitive decline is a significant issue involving several domains of cognitive abilities and which affects millions of older adults in the United States alone. Interventions that can help to remediate age-related reductions in cognitive functioning have the potential to greatly enhance the well-being and quality of life of a large portion of the aging population. Therefore, efforts designed to develop and evaluate new approaches to the remediation of age-related cognitive decline are a high priority.

### E.5. Data and Safety Monitoring Plan

#### E.5.a. Overview

This study will be overseen by a PI, Co-PI, Co-Is, a study statistician, and a designated study coordinator. In addition, the study will be reviewed at the University of Iowa IRB. The UI IRB reviews and approves research in accordance with Department of Health and Human Services (DHHS) regulations 45 CFR 46. In addition, for studies involving products regulated by the Food and Drug Administration (FDA), IRB-01 complies with the requirements set forth in 21 CFR 11, 21 CFR 50, 21 CFR 56, 21 CFR 312, 21 CFR 812, and 21 CFR 814, Subpart H. Following initial approval, all studies are also reviewed by the IRB on regular intervals (Continuing Review) appropriate to the degree of risk, but not less than once per year. Studies may also be selected for on-site review by the IRB based on the following criteria: 1) random selection from among all studies, 2) complex projects involving unusual levels or types of risks to subjects, 3) projects involving vulnerable populations, 4) projects conducted by an investigator who previously failed to comply with IRB determinations, or 5) projects where continuing review or reports from other sources have indicated that changes without IRB approval may have occurred. Note that because the proposed study is not an NIH-defined Phase III Clinical Trial and does not involve multiple sites, it will not be necessary to convene a Data and Safety Monitoring Board.

All participants in the study will be monitored for serious adverse events, protocol compliance, and adequate study progress. Every two weeks, the PI, Co-PI, and the study coordinator will review the following information in detail at the study meeting:

1) Participant recruitment rate;
2) Participant drop-out and the reasons for drop-out;
3) Serious Adverse Events
4) Participant compliance with the protocol;
5) Study coordinator questions or concerns.

Because the study investigators will be directly involved with this review, decisions about changes, modifications, or adaptations are decided and acted upon immediately. Protocols must be reviewed annually by the IRB at the University of Iowa. Reports must be generated about the study progress. Failure to complete these reports results in the suspension of the study.

**E.5.b. Policies and Procedures**

E.5.b.1. Enrollment and Consent Process

In order to take part in the study, each study participant must successfully complete a screening process, attend a baseline information session, and sign the informed consent before being enrolled and assigned to study conditions. This ensures that all participants meet all inclusion/exclusion criteria as stated in the study protocol and have provided informed consent. The participant will be given a copy of the informed consent, which includes written information on the nature of the study and the study interventions. After the informed consent is reviewed with the study participant, s/he will be given time to ask questions and decide whether or not s/he wishes to participate. After making sure the participant clearly understands the study procedures and agrees to follow them, they are asked to sign the informed consent. Participants will also be given information containing the names and telephone numbers of key study personnel, and will be encouraged to call at anytime should they have concerns or problems that might occur during the study.

E.5.b.2. Study Stopping Rules

Stopping Rules for this Study

Given the low risk nature of the cognitive training interventions and study procedures, we do not anticipate having to stop the study prematurely due to any safety concerns. Nevertheless, we have developed specific stopping rules to protect the safety of study participants. In the case of a serious adverse event (AE) [as defined by the FDA and the UI IRB], the study will be stopped and no further enrollment will take place until an investigation of the event has taken place by the PI or the co-investigators and the study coordinator. The FDA guidelines established for both anticipated and unanticipated Serious Adverse Events will be followed. A determination of the association of the adverse event with the study intervention will be made and appropriate modifications to the protocol will be made if an association is suspected. If protocol modifications to ensure the safety of future study participants cannot be made, the study will be terminated. The Program Officer from NIA will be notified of a study stoppage.

Other important issues to consider with regard to prematurely stopping a clinical trial pertain to conclusive evidence of futility or efficacy. Because the proposed study is designed to generate initial effect size estimates and is not adequately powered to determine efficacy, it is extremely unlikely that sufficient evidence will be generated to conclude that the cognitive training interventions are of no benefit, or that one is clearly associated with superior outcomes. Nevertheless, preliminary data analyses will be conducted on a semi-annual basis to review study progress and preliminary outcomes. If the investigative team finds evidence suggesting that early stoppage should be considered due to futility or conclusive evidence, then we will consult with the study sponsor.

Stopping Rules for the Individual Participant

For any reported adverse event, a study investigator must decide whether the event should be classified as serious or not. We will use standard definitions for Serious Adverse Events per FDA guidelines. In the unlikely event that an AE is thought to be study-related, the PI may elect to have the participant temporarily suspend study procedures. If the AE is not life-threatening and resolves itself, then the investigator may ask the participant to participate in a re-challenge (re-initiate the study intervention) during which time s/he will be closely monitored. The participant may refuse this at any time.

It should be noted that at any time during the study participation, if the participant stops taking part in the cognitive training program, we will ask that s/he continue follow-up in the study, even though they are no longer receiving the intervention.

E.5.b.3. Serious Adverse Events

As part of ongoing study monitoring, occurrence of any of the following events are required to be reported to the University of Iowa IRB:

1. Any unanticipated problems involving risks to subjects or others which occurs at the University of Iowa (UI) or that impacts UI subjects or conduct of the study;
2. A serious adverse drug event (either expected or unexpected) occurring in a UI study;
3. A serious adverse device effect (either anticipated or unanticipated) occurring in a UI subject;
4. An unanticipated serious adverse device effect occurring in a non-UI subject;
5. Receipt of new information (including risk or benefit) that may impact the willingness of subjects to participate or continue participation in the research study;
6. Any incidents of noncompliance with the federal regulations or the requirements or determinations of the IRB.

For purposes of study oversight and monitoring, we will use the FDA definition of serious adverse events (SAE). All SAE from both study sites will be systematically evaluated by the PI. In addition to reporting SAE (whether or not related to the intervention and study participation) to the IRB, all SAE will also be reported to NIA. The initial SAE report will be followed up with a completed SAE report that will be submitted to both the IRB and also to NIA. Participants who withdraw from a study voluntarily or whose participation is discontinued due to a SAE will continue to be monitored until a resolution or stabilization is reached, the SAE is determined to be clearly unrelated to study intervention or the SAE results in death. A summary of SAE that occurred during the previous year will be included in the annual report that is submitted to NIA as well as to the continuing renewal application that is sent to the IRB.

Following review of any SAE that are submitted on behalf of the study, the IRB shall take whatever action(s) it deems appropriate. These actions may include but are not limited to:

a) Modification of the protocol;
b) Modification of the consent form document;
c) Modification to the timetable for continuing review requirements;
d) Suspension of new enrollment into the study;
e) Suspension of the study; or
f) Termination of the study.

Any studies that are suspended or terminated related to a Serious Adverse Event will be promptly reported to the NIA within 48 hours. Any studies that are suspended or terminated related to a non-Serious Adverse Event will be reported to the NIA within 15 days.

**Plans for Reporting the Temporary or Permanent Suspension of the Trial to the Grant Program Officer**

All unanticipated protocol suspensions are accomplished with a protocol modification. All protocol modifications are reviewed by the UI IRB. The program officer will be notified of the suspension. Suspensions related to a Serious Adverse Event will be reported to the UI IRB within 24 hours and to the designated program officer within 48 hours.

**Plans for Assuring Compliance with Requirements for the Reporting of Adverse Events**

All study staff will be trained by an investigator on the FDA and DSMP criteria and policies for a Serious Adverse Event (SAE). An SAE in a participant will be reported to the IRB within 24 hours from the time it is identified.

For any reported unusual or potentially intervention-related side effect, the study staff is instructed to collect as much information as possible and to report it to the program coordinator or her assistant while the participant is still on the telephone or present at the study site. The coordinator will, in turn, consult with the study PI or Co-PI and the participant.

## E.6. Statement on Inclusion of Women and Minorities

Due to the demographic characteristics of patients attending the UIHC PCC and the target age range (50+ years of age), we expect the majority of study participants to Caucasian, with about 58% being women. For example, the current racial and ethnic composition of patients attending the UIHC PCC is as about 95% Caucasian, 2% African American, 1% Hispanic or Latino, 1% other. Nevertheless, our investigative team has long been committed to underserved populations. Therefore, significant efforts will be taken to oversample members of racial and ethnic minority groups to reach targets that are approximately double the current hospital rates for these groups.

# *Targeted/Planned Enrollment Table*

**This report format should NOT be used for data collection from study participants.**

**Study Title: RCT of Two Speed of Processing Modes to Prevent Cognitive Decline in Older Adults**

**Total Planned Enrollment: 900**

| TARGETED/PLANNED ENROLLMENT: Number of Subjects | | | |
|---|---|---|---|
| **Ethnic Category** | **Sex/Gender** | | |
| | **Females** | **Males** | **Total** |
| Hispanic or Latino | 12 | 11 | 23 |
| Not Hispanic or Latino | 510 | 367 | 515 |
| Ethnic Category Total of All Subjects* | 522 | 378 | 900 |
| **Racial Categories** | | | |
| American Indian/Alaska Native | 4 | 3 | 7 |
| Asian | 8 | 6 | 14 |
| Native Hawaiian or Other Pacific Islander | 0 | 0 | 0 |
| Black or African American | 27 | 19 | 46 |
| White | 482 | 351 | 833 |
| Racial Categories: Total of All Subjects * | 521 | 379 | 900 |

## E.6. Statement on Inclusion of Children

Because the study is addressing interventions for age-related cognitive decline, we will not be enrolling any children in the proposed study.  As noted above, participation will be limited to those who are at least 50 years of age.

## BIBLIOGRAPHY AND LITERATURE CITED

Ball, KK, JD Edwards, & LA Ross. (2007). The impact of speed of processing training on cognitive and everyday functions. *Journal of Gerontology: Social Sciences*, 62B:19-31.

Ball, KK, BL Beard, DL Roenker, RL Miller, & DS Griggs. (1988). Age and visual search: expanding the useful field of view. *Journal of the Optical Society of America*, 5:2210-2219.

Ball, KK, DB Berch, KF Helmers, JB Jobe, MD Leveck, M Marsiske, JN Morris, GW Rebok, DM Smith, SL Tennstedt, FW Unverzagt, & SL Willis. (2002). Effects of cognitive training interventions with older adults: a randomized controlled trial. *JAMA*, 288:2271-2281.

Edwards, JD, VG Wadley, DE Vance, K Wood, DL Roenker, & KK Ball. (2005). The impact of speed of processing training on cognitive and everyday performance. *Aging & Mental Health*, 9:262-271.

Jobe, JB, DM Smith, KK Ball, SL Tennstedt, M Marsiske, SL Willis, GW Rebok, JN Morris, KF Helmers, MD Leveck, & K Kleinman. (2001). ACTIVE: a cognitive intervention trial to promote independence in older adults. *Controlled Clinical Trials*, 22:453-479.

Kessler RC, & DF Greenberg. (1981). *Linear Panel Analysis*. New York: Academic Press.

National Academy of Sciences. (2006). *Workshop on Identifying New Interventions to Extend Disability Decline in Elderly Populations*. Bethesda, MD: National Academies of Science. Last accessed on March 16, 2009. http://www.nia.nih.gov/NR/rdonlyres/AF0997F6-0C16-4A76-96C0-D3780F00E6D4/5387/IDNewInterventionstoExtendDisabilityDecline2006Sep.pdf.

Rosnick, CB, BJ Small, AB Graves, & JA Mortimer. (2004). The association between health and cognitive performance in a population-based study of older adults: the Charlotte County Healthy Aging Study (CCHAS). *Aging Neuropsychology & Cognition*, 11:89-99

Salthouse, T. (1996). The processing-speed theory of adult age differences in cognition. *Psychological Review*, 103:403-428.

Salthouse, T. (2009). When does age-related cognitive decline begin? *Neurobiology & Aging*, 30:507-514.

Willis, SL, SL Tennstedt, M Marsiske, KK Ball, J Elias, K Mann-Koepke, JN Morris, GW Rebok, FW Unverzagt, A Stoddard, & E Wright. (2006). Long-term effects of cognitive training on everyday functional outcomes in older adults. *JAMA*, 296:2805-2814.

Wolinsky, FD, FW Unverzagt, DM Smith, R Jones, E Wright, & S Tennstedt. (2006a). The effects of the ACTIVE cognitive training interventions on clinically relevant declines in health-related quality of life. *Journal of Gerontology: Social Sciences*, 61B:S281-S287.

Wolinsky, FD, FW Unverzagt, DM Smith, R Jones, A Stoddard, & S Tennstedt. (2006b). The ACTIVE cognitive training trial and health-related quality of life: protection that lasts for five years. *Journal of Gerontology: Medical Sciences*, 61A:1324-1329.

Wolinsky, FD, HW Mahncke, M Kosinksi, FW Unverzagt, DM Smith, R Jones, A Stoddard, & SL Tennstedt. (2009a). The ACTIVE cognitive training trial and predicted medical expenditures. *BMC Health Services Research*, revised and resubmitted.

Wolinsky, FD, MW Vander Weg, R Martin, FW Unverzagt, KK Ball, R Jones, & SL Tennstedt. (2009b). The effect of speed of processing training on depressive symptoms in ACTIVE. *Journal of Gerontology: Medical Sciences*, 64A:468-472.

Wolinsky, FD, HW Mahncke, MW Vander Weg, R Martin, FW Unverzagt, KK Ball, R Jones, & SL. Tennstedt. (2009c). The ACTIVE cognitive training interventions and the onset of and recovery from suspected clinical depression. *Journal of Gerontology: Psychological Sciences*, revised and resubmitted.

Wolinsky, FD, HW Mahncke, MW Vander Weg, R Martin, FW Unverzagt, KK Ball, R Jones, & SL Tennstedt. (2009d). Speed of processing training improves self-rated health in older adults: enduring effects observed in the multi-site ACTIVE study. *Journal of Gerontology: Medical Sciences*, in review.

Wolinsky, FD, MW Vander Weg, R Martin, FW Unverzagt, SL Willis, M Marsiske, GW Rebok, JN Morris, KK Ball, & SL Tennstedt. (2009e). Cognitive training improves internal locus of control among older adults. *Journal of Gerontology: Social Sciences*, in review.

PositScience®
Your brain will thank you.™

March 23, 2009

Fredric D. Wolinsky, Ph.D.
The John W. Colloton Chair
College of Public Health
The University of Iowa
200 Hawkins Drive, GH-E205
Iowa City, Iowa 52242

Dear Fred:

I write as the Founder and Chief Scientific Officer of Posit Science Corporation to express my full support for your NIH Challenge Grant proposal ("RCT of Two Speed of Processing Modes to Prevent Cognitive Decline in Older Adults") to use Road Tour$^{TM}$ in a three group randomized controlled trial of 900 patients attending the Primary Care Clinics at the University of Iowa. Clearly, the long-term goal of your research program is to utilize speed of processing interventions like Road Tour$^{TM}$ more broadly to facilitate well-being and quality of life among all older adults. This is an important and noble pursuit.

Your proposal builds nicely on the nearly two decades of previous studies conducted by Dr. Karlene Ball and colleagues. In particular, it follows well on the recent results of the NIH-funded ACTIVE study demonstrating improvements in processing speed and also logically progresses beyond your own innovative analyses showing profound protective effects of speed of processing on (1) avoiding global declines in health-related quality of life, (2) reducing predicted medical care costs, (3) avoiding clinically relevant increases in depressive symptoms, (4) protecting against the onset of clinical depression, and (5) improving self-rated health and internal locus of control.

It is my honor, therefore, to provide you with 900 licenses of Road Tour$^{TM}$ for use in your NIH Challenge Grant at no cost whatsoever. Given the current per user cost of Road Tour$^{TM}$ at $395 each, this represents a $355,500 in-kind contribution from Posit Science Corporation. Furthermore, we will provide you full and complete encrypted access to our secure server to retrieve the automated downloads of time spent on Road Tour$^{TM}$ and the performance test data for monitoring your participants' progress within the study. And of course, we stand ready to provide whatever other assistance you may need in this important study.

Finally, I pledge that when your study has concluded, and your work is seen as a wonderful scientific success and all of your hypotheses have been supported, the federal government's investment in this science will be reflected in Posit Science's commitment to work with agencies at the federal government to make the program available for wide-scale implementation at only a fraction of the current per-user cost.

Fred, we at Posit Science are delighted to enthusiastically support your proposed NIH Challenge Grant. We are prepared to help in any way that we can.

Sincerely,

Michael Merzenich

Michael M. Merzenich, Ph.D.
Founder & Chief Scientific Officer
Posit Science Corporation

Professor Emeritus & Co-Director of the Coleman Memorial Laboratory
Keck Center for Integrative Neurosciences, University of California at San Francisco

Posit Science 225 Bush Street 7th floor San Francisco CA 94104 main 415 394 3100 fax 415 986 2829
www.PositScience.com

## Resource Sharing Plan

We will make all of the data obtained from this study publicly available as an anonymous, de-identified data set within three years of the project completion, and will deposit that data with the Inter-University Consortium for Political and Social Research (ICPSR) at the University of Michigan. In constructing this HIPPA compliant, de-identified data set for public use, we will follow the *Guide to Social Science Data Preparation and Archiving*, 3rd edition, published by the ICPSR in 2005.

# PHS 398 Checklist

OMB Number: 0925-0001
Expiration Date: 9/30/2007

## 1. Application Type:

From SF 424 (R&R) Cover Page. The responses provided on the R&R cover page are repeated here for your reference, as you answer the questions that are specific to the PHS398.

* Type of Application:

☒ New ☐ Resubmission ☐ Renewal ☐ Continuation ☐ Revision

Federal Identifier:

## 2. Change of Investigator / Change of Institution Questions

☐ Change of principal investigator / program director

Name of former principal investigator / program director:

Prefix:
* First Name:
Middle Name:
* Last Name:
Suffix:

☐ Change of Grantee Institution

* Name of former institution:

## 3. Inventions and Patents (For renewal applications only)

* Inventions and Patents: Yes ☐ No ☐

If the answer is "Yes" then please answer the following:

* Previously Reported: Yes ☐ No ☐



Tracking Number:GRANT10250732 Funding Opportunity Number:RFA-OD-09-003 Received Date:2009-04-15T09:22:36-04:00

OMB Number. 0925-0001
Expiration Date: 9/30/2007

## 4. * Program Income

Is program income anticipated during the periods for which the grant support is requested?

☐ Yes ☒ No

If you checked "yes" above (indicating that program income is anticipated), then use the format below to reflect the amount and source(s). Otherwise, leave this section blank.

| *Budget Period | *Anticipated Amount ($) | *Source(s) |
|---|---|---|
| | | |
| | | |
| | | |
| | | |
| | | |

## 5. Assurances/Certifications (see instructions)

In agreeing to the assurances/certification section 18 on the SF424 (R&R) form, the authorized organizational representative agrees to comply with the policies, assurances and/or certifications listed in the agency's application guide, when applicable. Descriptions of individual assurances/certifications are provided at: http://grants.nih.gov/grants/funding/424

*If unable to certify compliance* , where applicable, provide an explanation and attach below.

Explanation: [ ] Add Attachment | Delete Attachment | View Attachment



Tracking Number:GRANT10250732 Funding Opportunity Number:RFA-OD-09-003 Received Date:2009-04-15T09:22:36-04:00
